# Joint Genomic and Proteomic Analysis Identifies Meta-Trait Characteristics of Virulent and Non-virulent *Staphylococcus aureus* Strains

**DOI:** 10.3389/fcimb.2018.00313

**Published:** 2018-09-06

**Authors:** Emilia A. Bonar, Michal Bukowski, Marcin Hydzik, Urszula Jankowska, Sylwia Kedracka-Krok, Magdalena Groborz, Grzegorz Dubin, Viktoria Akkerboom, Jacek Miedzobrodzki, Artur J. Sabat, Alexander W. Friedrich, Benedykt Wladyka

**Affiliations:** ^1^Department of Analytical Biochemistry, Faculty of Biochemistry, Biophysics and Biotechnology, Jagiellonian University, Krakow, Poland; ^2^Malopolska Centre of Biotechnology, Jagiellonian University, Krakow, Poland; ^3^Department of Physical Biochemistry, Faculty of Biochemistry, Biophysics and Biotechnology, Jagiellonian University, Krakow, Poland; ^4^Department of Microbiology, Faculty of Biochemistry, Biophysics and Biotechnology, Jagiellonian University, Krakow, Poland; ^5^Department of Medical Microbiology, University Medical Center Groningen, University of Groningen, Groningen, Netherlands

**Keywords:** genome, genomics, pathogen, proteome, proteomics, *Staphylococcus aureus*, virulence

## Abstract

*Staphylococcus aureus* is an opportunistic pathogen of humans and warm-blooded animals and presents a growing threat in terms of multi-drug resistance. Despite numerous studies, the basis of staphylococcal virulence and switching between commensal and pathogenic phenotypes is not fully understood. Using genomics, we show here that *S. aureus* strains exhibiting virulent (VIR) and non-virulent (NVIR) phenotypes in a chicken embryo infection model genetically fall into two separate groups, with the VIR group being much more cohesive than the NVIR group. Significantly, the genes encoding known staphylococcal virulence factors, such as clumping factors, are either found in different allelic variants in the genomes of NVIR strains (compared to VIR strains) or are inactive pseudogenes. Moreover, the pyruvate carboxylase and gamma-aminobutyrate permease genes, which were previously linked with virulence, are pseudogenized in NVIR strain ch22. Further, we use comprehensive proteomics tools to characterize strains that show opposing phenotypes in a chicken embryo virulence model. VIR strain CH21 had an elevated level of diapolycopene oxygenase involved in staphyloxanthin production (protection against free radicals) and expressed a higher level of immunoglobulin-binding protein Sbi on its surface compared to NVIR strain ch22. Furthermore, joint genomic and proteomic approaches linked the elevated production of superoxide dismutase and DNA-binding protein by NVIR strain ch22 with gene duplications.

## Introduction

The genetic determinants and protein effectors that are responsible for the virulence of *Staphylococcus aureus* escape our full understanding despite a number of comprehensive studies. In homeostasis, *S. aureus* coexists with its host without distinguished adverse effects. However, in an imbalanced state, the nature of which is poorly understood, this opportunistic pathogen may cause infection and pose a significant health threat. Thus, the Janus-face bacteria constantly balances commensal and virulent phenotypes, coping with different levels of host defenses (Rasigade and Vandenesch, 2014). Indeed, it was recently demonstrated that within the same clonal complex, phenotypic differences may be linked with the severity of infections. Moreover, factors correlated with high pathogenicity in the group of genetically related *S. aureus* had little effect on the mortality rates associated with infections caused by bacteria from other clonal complexes (Recker et al., 2017). This finding indicates both the genetic and phenotypic basis of staphylococcal virulence. Aside from maintaining host/pathogen balance in a single host species, staphylococci have been demonstrated to switch between animal and human hosts. Such switching is associated with the exchange of host-specific virulence factors that are responsible for colonization and spread (Lowder et al., 2009). This plasticity significantly complicates studies on virulence determinants, especially in terms of likely human specific factors that can be experimentally tested exclusively in animal models. Genetic methods have been successfully used to predict antibiotic resistance with high credibility and the recent advent of massive parallel sequencing promises clinical utility (Aanensen et al., 2016). However, only a few genetic markers, whose mechanism of action has been determined at the molecular level, have been convincingly linked with successful colonization and virulence [e.g., *a*rginine *c*atabolism *m*obile *e*lement, ACME (Diep et al., 2008; Thurlow et al., 2013), exfoliative toxins (Bukowski et al., 2010)]. Other genetic markers, based on statistical analysis of whole genome sequencing and DNA microarray assays data, were pointed to positively correlate with severity of infections, including bacteremia and infective endocarditis (Gill et al., 2011; Calderwood et al., 2014; Bouchiat et al., 2015). Nevertheless, a palette of genetic predictors of staphylococcal virulence is still limited. A number of studies have used proteomics approaches to try to identify the protein effectors of virulent phenotypes (Bonar et al., 2015). Earlier, we compared exoproteomes in a set of strains exhibiting high and low virulence in a chicken embryo infection model. Despite the high heterogeneity of the analyzed proteomes, we were nevertheless able to identify alpha-hemolysin and bifunctional autolysin as indicators of virulence, whereas glutamylendopeptidase production was characteristic of non-virulent strains (Bonar et al., 2016). This prior study, however, did not take into account surface-attached proteins, which may contain additional virulence factors. The intracellular proteome was not taken into consideration either, but it may potentially contain regulatory proteins.

However, another source of information on staphylococcal virulence factors traditionally comes from the investigation of knock-out and knock-in strains devoid of or supplemented with tested factors and subsequently challenged in animal models (Kim et al., 2014b). Unfortunately, a lack of appropriate models has detrimentally affected the results (Polakowska et al., 2012). Moreover, this approach does not allow to distinguish “true” virulence factors from those affecting the overall fitness of bacteria.

Given the limitations of these current approaches, here, we designed and applied a new, combined workflow to successfully identify virulence- and commensalism-related meta-traits within the genomes and proteomes of virulent (VIR) and non-virulent (NVIR) *S. aureus* strains. Two belonging to the same sequence type wild-type strains that have been well-characterized in terms of virulence in an *in vivo* model were compared and contrasted using a combined genomic and proteomic methodology. We show that the non-virulent strain ch22 is characterized by a more complex exoproteome than its virulent counterpart CH21. This finding is associated with the smaller genome of CH21 than ch22. Interestingly, CH21 is not characterized by the production of any classical virulence factors compared to ch22. It is rather the combined differential expression of multiple factors that determines the virulence of CH21; the rationale behind this conclusion is discussed in our communication.

## Materials and methods

### Bacterial strains and growth conditions

Poultry-isolated *S. aureus* strains exhibiting either high (CH3, CH5, CH9, CH21, and CH23) or low (ch22, ch24, pa3, and ph2) virulence (VIR and NVIR, respectively) in a chicken embryo experimental infection model were used in the study (Supplementary Table 1). Strain origin and general genetic and phenotypic characteristics, including basic phylogenetic relationships and virulence, were described previously (Lowder et al., 2009; Polakowska et al., 2012; Bonar et al., 2016). The bacteria were cultured in tryptic soy broth (TSB) for 16 h at 37°C with vigorous shaking unless indicated otherwise.

### Genome sequencing and assembly

#### Whole genome sequencing

Genomic DNA was isolated using a DNeasy Blood and Tissue Kit (Qiagen) from an overnight culture derived from a single colony. Purified DNA was quantified with a Qubit 2.0 Fluorometer (Life Technologies). Whole genome sequencing was performed using an Illumina MiSeq system with DNA fragment libraries prepared using a Nextera XT v3 kit (Illumina) according to the manufacturer's protocol. The samples were sequenced to obtain a minimum of 100-fold coverage. Reads were *de novo* assembled into contigs using CLC Genomics Workbench (version 8.5.1). Contigs were ordered on a template of the *S. aureus* ED98 complete chromosome sequence (GenBank CP001781.1) using self-developed Python scripts, which utilized nucleotide BLAST from the NCBI BLAST+ toolkit [version 2.3.0 (Camacho et al., 2009)]. The complete genomic sequences of the CH21 and ch22 strains were obtained by closing the remaining gaps using PCR amplification and Sanger sequencing. Automated genome annotation was performed using the NCBI Prokaryotic Genome Annotation Pipeline (http://www.ncbi.nlm.nih.gov/genome/annotation_prok/). The sequences were deposited in GenBank with the accession numbers: CH3, MOYG00000000; CH5, MSGQ00000000; CH9, MOYH00000000; CH21, CP017804, CP017804, CP017806; ch22, CP017807, CP017808, CP017809; ch23, MOYI00000000; ch24, MOYJ00000000; pa3, MOXP00000000; ph2, MOYK00000000. Detailed information may be found in the Supplementary Table 1.

#### Identification of mobile genetic elements (MGEs)

The contigs that did not match the chromosome sequence were examined for possible plasmid origin by evaluating their similarity to existing plasmids or fragments thereof. Whole genomic sequences were examined for known staphylococcal phages and pathogenicity islands using an exhaustive set of reference sequences obtained from GenBank (Supplementary Table 2). Short sequence fragments of at least 1 kb showing similarity (but not identical) to known phages and pathogenicity islands were classified as putatively novel.

#### Construction of phylogenetic trees

Phylogenetic trees of the identified prophages and pathogenicity islands were constructed using CLC Main Workbench (version 7.7.2) and the k-mer based tree construction method with the Neighbor Join algorithm (k-mer size 15; distance measure: fractional common k-mer count). SNP analysis was performed using the CSI Phylogeny 1.4 server (Kaas et al., 2014). As the chromosome sequences were submitted to the server minimum depth at SNP positions, minimum relative depth at SNP positions, minimum distance between SNPs and minimum SNP quality as input parameters were disabled during analysis. The read mapping quality was set to minimum 25 and the z-score to 1.96. The maximum likelihood tree produced by CSI Phylogeny 1.4 server was visualized in MEGA6 software (Tamura et al., 2013).

#### *In silico* MLST and ST5 group phylogenetic analysis

A pipeline was developed in Python 3 for *in silico* MLST and phylogenetic analysis of strains belonging to ST5 group. All 8,688 *S. aureus* genomes, complete genomic sequences as well as shotgun sequencing results, were fetch from NCBI GenBank database (ftp://ftp.ncbi.nlm.nih.gov/genomes/genbank/bacteria/, accessed on 2018-07-06). 289 genomes which did not provide a cross-reference to NCBI Biosample database were omitted. Loci profiles from BISGs database were used to classify the genomes according to their sequence type (https://pubmlst.org/saureus/, accessed on 2018-07-06 (Jolley and Maiden, 2010)]. Loci sequences used for *S. aureus* MLST typing were obtained from USA300 strain genomic sequence (accession version CP014420.1) and used as queries for nucleotide BLAST search of the remaining genomes with *E*-value threshold 0.0001 and required query coverage 100% (ver. 2.7.1+ (Camacho et al., 2009). For 2,129 assemblies, classified as ST5, cross-references to NCBI Biosample database (ftp://ftp.ncbi.nlm.nih.gov/biosample/, accessed on 2018-07-06) were used to obtain information on the host, year of isolation and country of origin (attributes of “harmonized_name” property equal to “host,” “geo_loc_name,” and “collection_date,” respectively). 1,635 assemblies contained such information. The information on the reference strains ED98 and N315, missing in NCBI Biosample database, was added manually. These genomes were search with nucleotide BLAST with *E*-value threshold 0.0001 and minimal query coverage 95% for loci sequences obtained from N315 strain genome (accession version BA000018.3), which were utilized before for ST5 phylogenetic analysis by Nubel et al. (2008). However, to avoid false positive hits, only loci of length equal or greater than 400 bp were selected (in total 97 of 126, Supplementary Table 10). For a significant over-representation of strains isolated from human host and originating from the USA (1,248 of 1,474, 85%), a random sample of size 50 was obtained for this group. Selected in such a way 279 strains together with other six being analyzed in this research and belonging to ST5 group (Supplementary Table 11) were subjected to phylogenetic analysis. For every strain the sequences corresponding to the aforementioned N315 strain loci were ordered, concatenated and aligned using Clustal Omega with default parameters [ver. 1.2.1 (Sievers and Higgins, 2014)]. Subsequently every column containing a gap was removed from the alignment, which shortened it from 44,295 to 43,872 bp. The alignment was converted to PHYLIP format) and used to create a phylogenetic tree with RAxML [ver. 8.2.9 (Stamatakis, 2014)] based on GTRGAMMAI model [general time-reversible, GTR (Tavaré, 1986)]. The tree was subsequently imported to CLC Main Workbench (Qiagen, CLC Bio) together with data obtained from Biosample database, as metadata, and visualized.

#### Analysis of coding sequences and putative promoters

Self-developed Python scripts utilizing nucleotide and protein BLAST from NCBI BLAST+ toolkit (version 2.3.0) and muscle [version 3.8.31 (Edgar, 2004)] were used for creating multiple sequence alignments to analyze coding sequences and putative promoter regions. Annotated coding sequences were extracted from the analyzed genomes together with 200 bp upstream fragments containing the putative promoters. Translations of the coding sequences were clustered with a similarity threshold of 90%. All identical protein sequences within a cluster were designated as a single allele. The multiple sequence alignments of different alleles and promoter sequences within each cluster were scanned for differentiating columns.

#### Visual genome comparison with BRIG

The nine genomes, published here, and the genome of N315 strain as a human reference of ST5 were compared to ED98 genome using BRIG tool (Alikhan et al., 2011). The identity thresholds were set to 98 and 95% and ED98 mobile genetic elements, prophages φAv1, φAvβ, the pathogenicity island SaPIAv, and plasmids pAvX, pT181, and pAvY were annotated.

#### Whole genome general comparison of CH21 and ch22 strains

Continuous similar sequence segments were identified using nucleotide BLAST. Most segments were separated by short insertions or duplications. Similar sequence segments and their equivalence between strains were visualized using Circos [version 0.69-3 (Krzywinski et al., 2009)].

### Proteomic analysis

#### Exoproteome analysis

For comparative analysis of the exoproteomes of the CH21 and ch22 strains, triplicates of cleared culture fluids were handled as described previously (Bonar et al., 2016). To assure no contamination with intracellular proteins, the supernatants were passed through a 0.22-μm PVDF filter. Then, the proteins were precipitated with an equal volume of 20% (w/v) trichloroacetic acid in acetone and recovered by centrifugation. The pellet was washed with acetone and air dried. The samples were dissolved in lysis buffer (30 mM TrisHCl pH 8.5 containing 7 M urea, 2 M thiourea and 4% CHAPS). The protein samples were labeled with spectrally resolvable fluorescent G-dyes (NH Dye AGNOSTICS GmbH) and subjected to two-dimensional difference gel electrophoresis [2D DIGE; (Alban et al., 2003; Timms and Cramer, 2008; Minden et al., 2009)]. Isoelectrofocusing (IEF) was performed using 17-cm immobilized pH (3–10) gradient strips and Protean IEF Cell (Bio-Rad). Proteins were separated in the second dimension using an 12% acrylamide gel according to the Laemmli method (Laemmli, 1970). The gels were scanned using Typhoon Trio + (GE), and images were analyzed using Image Quant v.7.0 and DeCyder 2D software v.7.2 (GE). Protein spots were considered as differentiating if the standardized average spot volume ratio exceeds 1.5-fold at the 95% confidence level (Student's *t*-test *p*-value < 0.05). Subsequently, the gels were silver stained (Shevchenko et al., 1996). The differentiating spots were excised and destained by several subsequent washes in 25% and 50% (v/v) acetonitrile (ACN) in 25 mM ammonium bicarbonate buffer (NH_4_HCO_3_), pH 8.0 at 37°C. The gel fragments were dehydrated in ACN, dried using a vacuum concentrator, and rehydrated using trypsin solution (10 ng/μl in 25 mM NH_4_HCO_3_, pH 8.0), and digestion was carried out overnight at 37°C. Peptides were extracted by sonication, dehydrated in ACN and dried using a vacuum concentrator. Samples were suspended in 2% (v/v) ACN in water containing 0.05% (v/v) trifluoroacetic acid (TFA) and separated using an UltiMate 3000RS LCnanoSystem (Dionex). Peptides were analyzed on a coupled MicrOTOF-QII mass spectrometer (Bruker) equipped with an Apollo Source ESInanosprayer with low-flow nebulizer. Raw data werepre-processed with Data Analysis 4.0 software (Bruker, Germany) into Mascot Generic format. The SwissProt non-redundant protein database taxonomically restricted to *Firmicutes* (gram-positive bacteria) was queried with the obtained peak lists using an in-house Mascot server. Additionally, an in-house prepared database constructed based on the full genome sequences of CH21 and ch22 obtained in this study was used. Only identifications with a score value over 100 were considered relevant for further analysis. If more than a single protein was identified in particular spot, only hits scoring over 50% of the highest scoring protein were considered in further analysis.

#### Analysis of intracellular proteome

For comparative analysis of the intracellular proteomes of strains CH21 and ch22, 3 ml of overnight cultures (OD_600_ ~ 12) was used in triplicate. The cells were collected by centrifugation, washed three times with 10 mM TrisHCl pH 8.0 and suspended in 1 ml of Tri Reagent (Sigma Aldrich), transferred to Lysis Matrix Tubes (MP Biomedicals) and disrupted with Precellys 24 Homogenizer (Bertin Instruments). Lysates were clarified by centrifugation, and the protein phase was isolated according to the Tri Reagent protocol. The obtained protein pellet was suspended in lysis buffer and analyzed by 2D DIGE and MS as described for exoproteins.

#### Surface proteome analysis

Proteolytic “shaving” was used to compare surface proteomes (surfacomes) of strains CH21 and ch22. All samples were analyzed in biological triplicates. Bacteria were cultured overnight in TSB at 37°C with agitation. The fresh medium was inoculated with the overnight culture at 1:100 dilution and cultured until OD_600_ reached 1. The cultures were divided into two samples, experimental and control, based on the method of Solis et al. (2010) to control for contamination by cytoplasmic proteins resulting from unspecific cell lysis. Bacteria were collected by centrifugation, washed with phosphate buffered saline (PBS) and suspended in 30% sucrose in PBS. Experimental samples were incubated with trypsin [1 μg/μl; “Gold, MS Grade” (Promega)] for 30 min at 37°C with gentle agitation, clarified by centrifugation and filtered through a 0.22-μm PVDF filter. The control sample was handled as above save that trypsin was not added during the initial incubation. The control sample was treated with trypsin (1 μg/μl) only after filtering (conditions as above). All samples were reduced with DTT and alkylated with idoacetamide, digested overnight with trypsin 0.2 μg/μl (Biocentrum) at 37°C, supplemented with 0.5% TFA and 5% ACN (final concentration), cleaned using Pierce C18 Spin columns (Thermo Fisher Scientific) and vacuum dried. Samples were analyzed by MS as described for exoproteins, save score values over 50 were considered relevant.

## Results and discussion

### Overall genomic characteristics of virulent and non-virulent strains of *S. Aureus*

**Figure 1:**
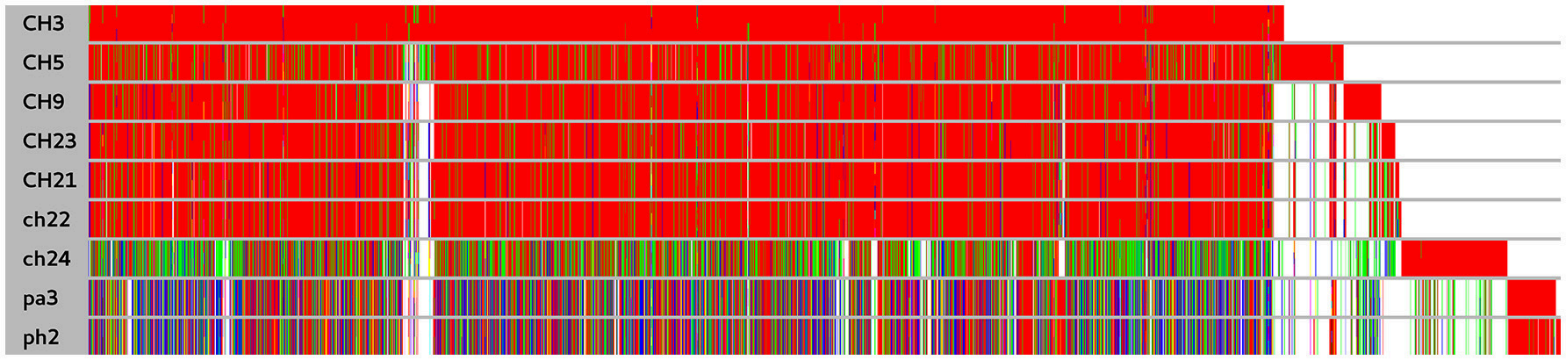
Comparison of protein clusters among nine *S. aureus* strains. Clusters were obtained for annotated coding sequences at threshold of 90% similarity. Within a cluster identical sequences are denoted by the same color. White denotes the absence of an ortholog. Long white gaps at the end of the plot correspond to the absence of different mobile genetic elements such as plasmids. The cluster order is related to the order of coding sequence occurrence in the genome of ED98.

**Figure 2:**
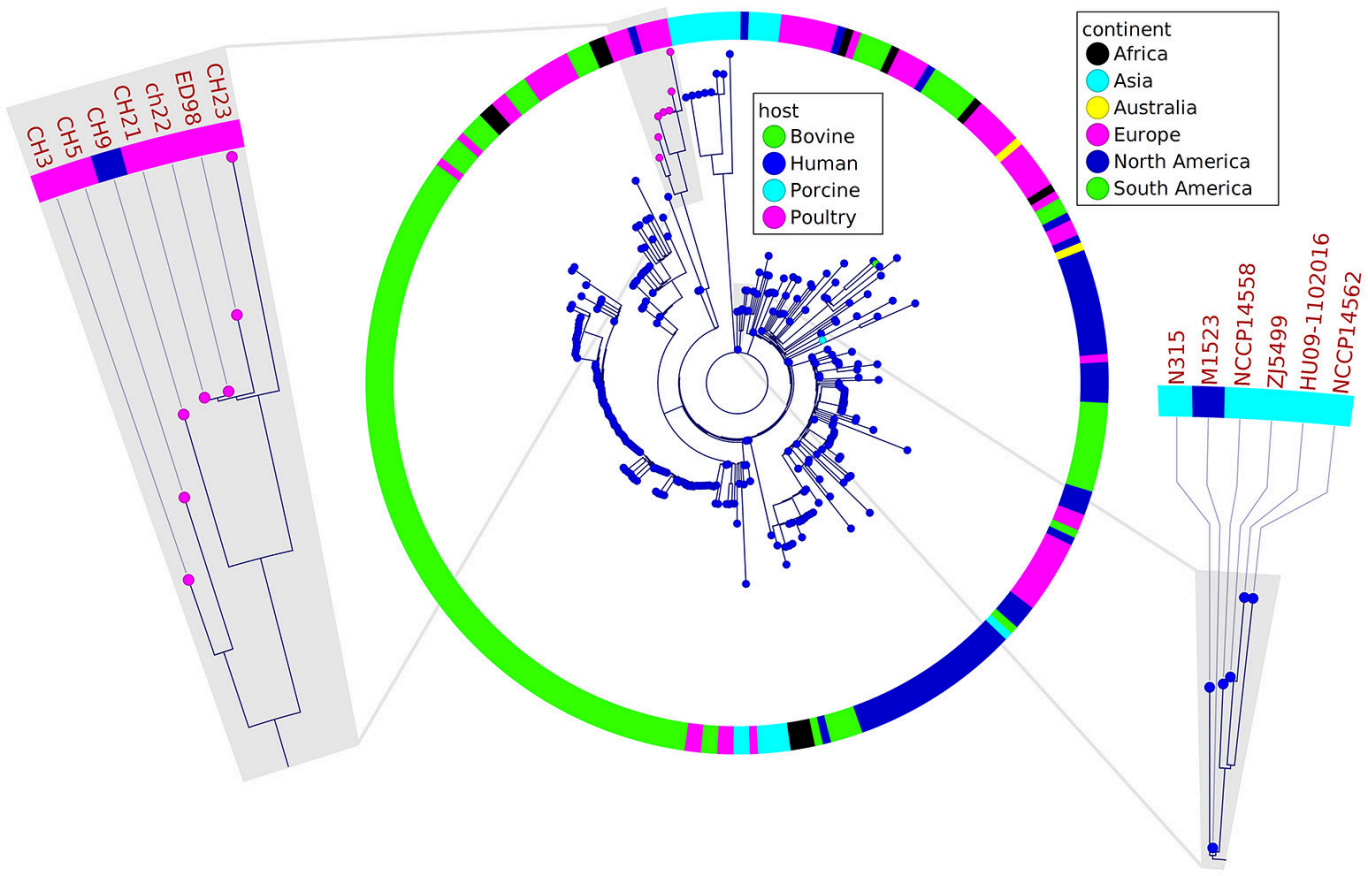
Phylogenetic relatedness of analyzed poultry strains to a representative group of other strains belonging to ST5 group determined by the analysis of 97 different loci, 44 kb of sequence in total. The poultry-associated strains comprise a distinctive phylogenetic group. Markedly, CH21 and ch22 strains are indistinguishable. Fragments of the tree containing reference strains ED98 and N315 for poultry- and human-origin strains, respectively, are magnified.

**Figure 3:**
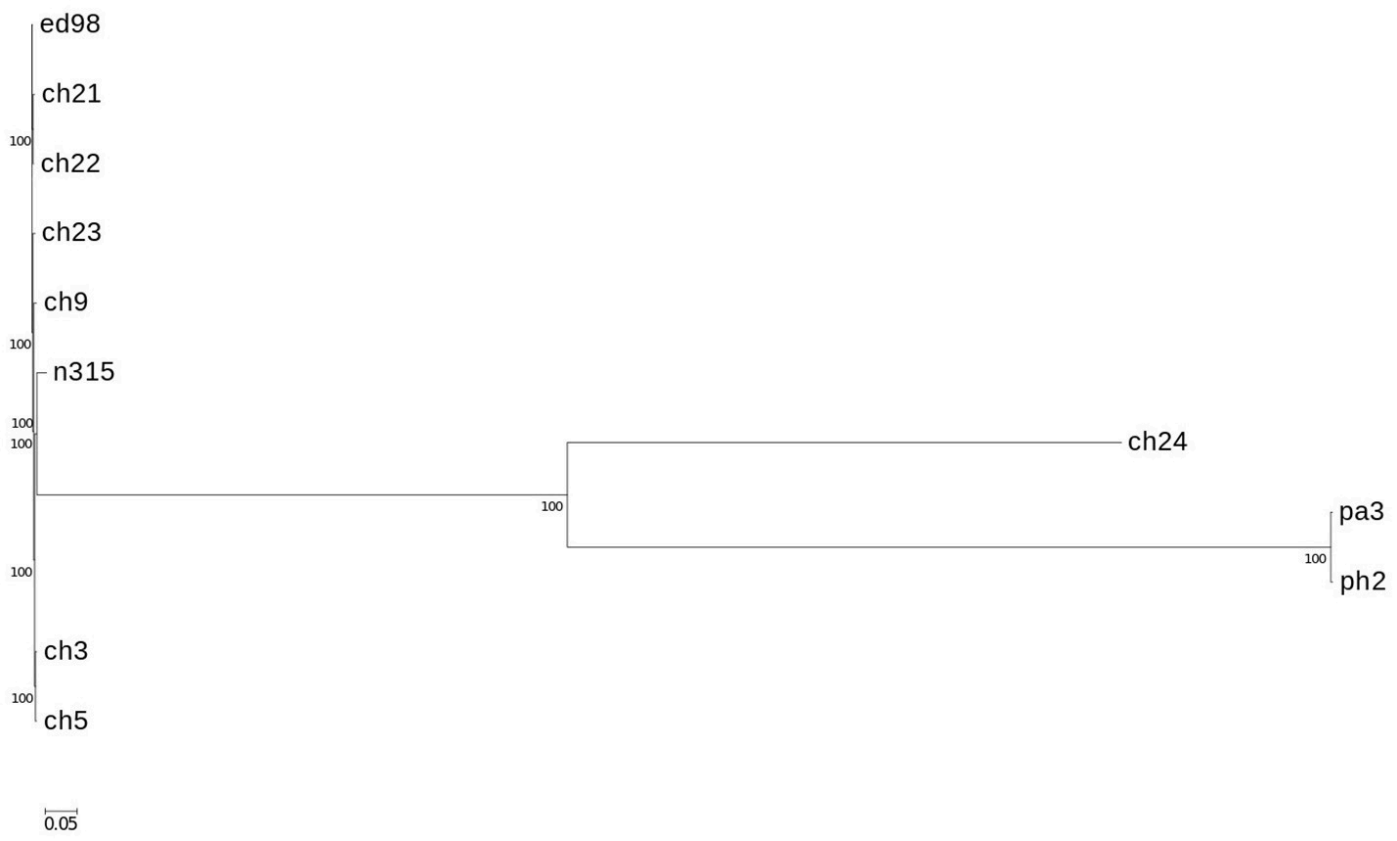
Phylogenetic relationship of strains mapped against reference type strain ED98. Phylogenetic maximum likelihood tree constructed on the basis of SNPs was obtained by CSI phylogeny 1.4. A confidence score ranging from 0 to 1 (×100 during visualization in MEGA6 software) was calculated for robustness evaluation of the nodes. The scale bar indicates the evolutionary distance between the sequences determined by 0.05 substitutions per nucleotide at the variable positions.

**Figure 4:**
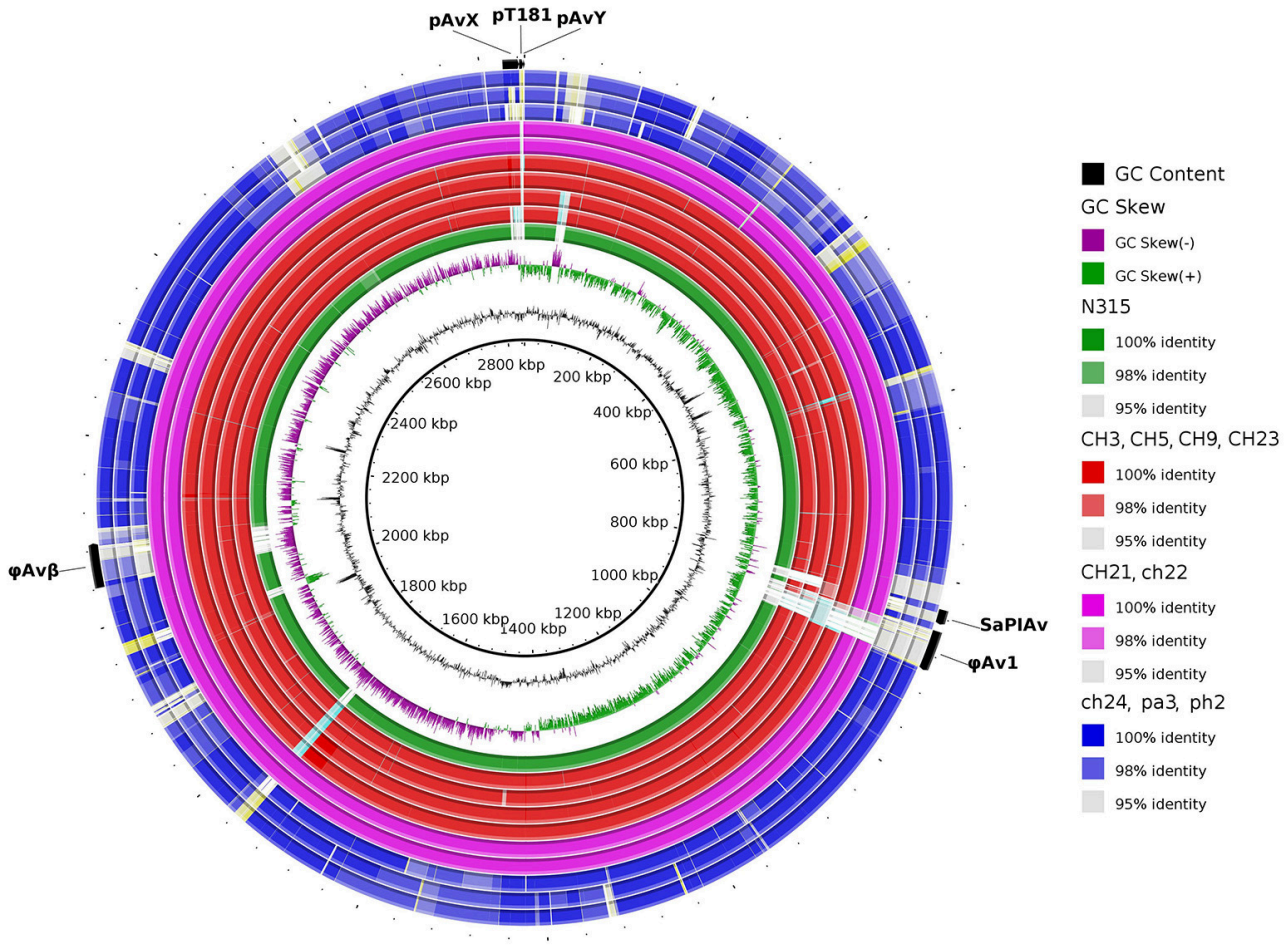
Visual comparison of genomes of poultry-origin *S. aureus* strains against a reference strain ED98. The group of virulent strains is genetically more homogenous when compared with the more diverse group of non-virulent strains. Particularly regarding indistinguishable CH21 and ch22 strains. Noticeably, mobile genetic elements such as prophages φAv1, φAvβ and pathogenicity island SaPIAv seem to be characteristic for the ST5 poultry subgroup. Human-origin MRSA strain N315 belonging to ST5 was also included.

In a prior study, we evaluated the virulence in a chicken embryo infection model of a number of poultry originating strains of *Staphylococcus aureus* on a background of their genetic relationships. To determine the genomic basis of phenotypic differences between the selected strains, in this study, we obtained the genomic sequences of five highly virulent strains (CH3, CH5, CH9, CH21, and CH23) and four strains characterized by low virulence (ch22, ch24, pa3, and ph2). Two genomes (CH21 and ch22) were obtained in their complete form, whereas the remaining seven genomes were obtained in the form of ordered contigs. The overall characteristics of the obtained genomes are summarized in Supplementary Table 1. Genome size does not correlate with virulent phenotypes since all genomes, save that of strain ch22 (considered in more detail in the next section), have a similar size of ca. 2.8 Mbp. Furthermore, the overall number of genes and coding sequences does not correlate with virulent phenotypes and is comparable among the analyzed strains save for ch22. Significantly, the virulent phenotype correlates with the presence of phage φAvB (46,768 bp; 62 ORFs) and pAvY plasmid (1,442 bp, 1 ORF). All VIR strains carry the above genetic elements, whereas all NVIR strains, with the exception of ch22, are devoid of both of these elements. Apart from the above mobile genetic elements, we additionally identified a number of novel prophages and pathogenicity islands and several known plasmids, neither of which differentiated the VIR and NVIR groups (Supplementary Tables 3, 4; Supplementary Figures 1, 2). Another clear distinction differentiating the VIR and NVIR groups becomes apparent when ORFs longer than 165 nucleotides are extracted and clustered according to protein sequence similarities above 90% (Figure 1). When the core genome of all nine strains is considered, only approximately one-quarter of genes fall within such defined clusters, which clearly demonstrates significant genetic differences among strains. Interestingly, the percentage of genes clustered with the above criteria among the core genomes of the VIR and NVIR groups is much higher at 72 and 42%, respectively, demonstrating the closer relatedness of strains within both groups than between groups. Such analysis also shows that the VIR group is more homogenous than the NVIR group (Table 1). When compared to human-origin strains of sequence type 5 (ST5), the ST5 poultry-origin strains comprise a clear evolutionary subgroup. This phylogenetic relationship based on more in depth analysis of genome-extracted 97 different loci (Nubel et al., 2008) places poultry-origin ST5 strains on a separate branch of the tree regardless to geographical origin. Interestingly, based on this analysis CH21 and ch22 strains are still genetically identical (Figure 2). Markedly, the whole genome analysis confirmed their high genetic resemblance by revealing only 85 single nucleotide polymorphisms (SNPs). The maximal SNP number among the whole poultry ST5 subgroup is 355, whereas the distance to the type strain N315 of human origin is higher (from 663 to 823 SNPs). Non-ST5 poultry strains analyzed in this study, all being non-virulent, are clearly more distant from the aforementioned ST5 subgroup by having from 19,384 to 21,127 SNPs. The ST692 strains pa3 and ph2, although isolated from different poultry hosts, respectively partridge and pheasant, are genetically highly similar to each other by displaying only 165 SNPs. The ST1 strain ch24 is highly distant from the rest of the analyzed strains by having from 19,384 to 20,875 SNPs (Figure 3; Supplementary Table 12). All these genetic similarities and differences are also visible when the analyzed genomes are visually compared to ED98 genome as a reference (Lowder et al., 2009). Notably, none of the non-ST5 strains contain mobile genetic element such as prophages φAv1, φAvβ and the pathogenicity island SaPIAv, which seem to be characteristic to the ST5 poultry subgroup (Figure 4).

**Table 1:** Number of clusters gathering open reading frames exhibiting over 90% protein sequence similarity in the studied group of *S. aureus* strains.

	**Total number of identified clusters**	**Number (percent) of identical clusters**
Pan-genome	3,307	1,277 (38.62)
Core-genome	2,317	586 (25.29)
VIR strains pan-genome	2,949	2,513 (85.22)
VIR strains core-genome	2,527	1,826 (72.26)
NVIR strains pan-genome	3,009	1,862 (61.88)
NVIR strains core-genome	2,471	1,050 (42.25)

### Genetic differences between virulent (CH21) and non-virulent (ch22) strains

**Figure 5:**
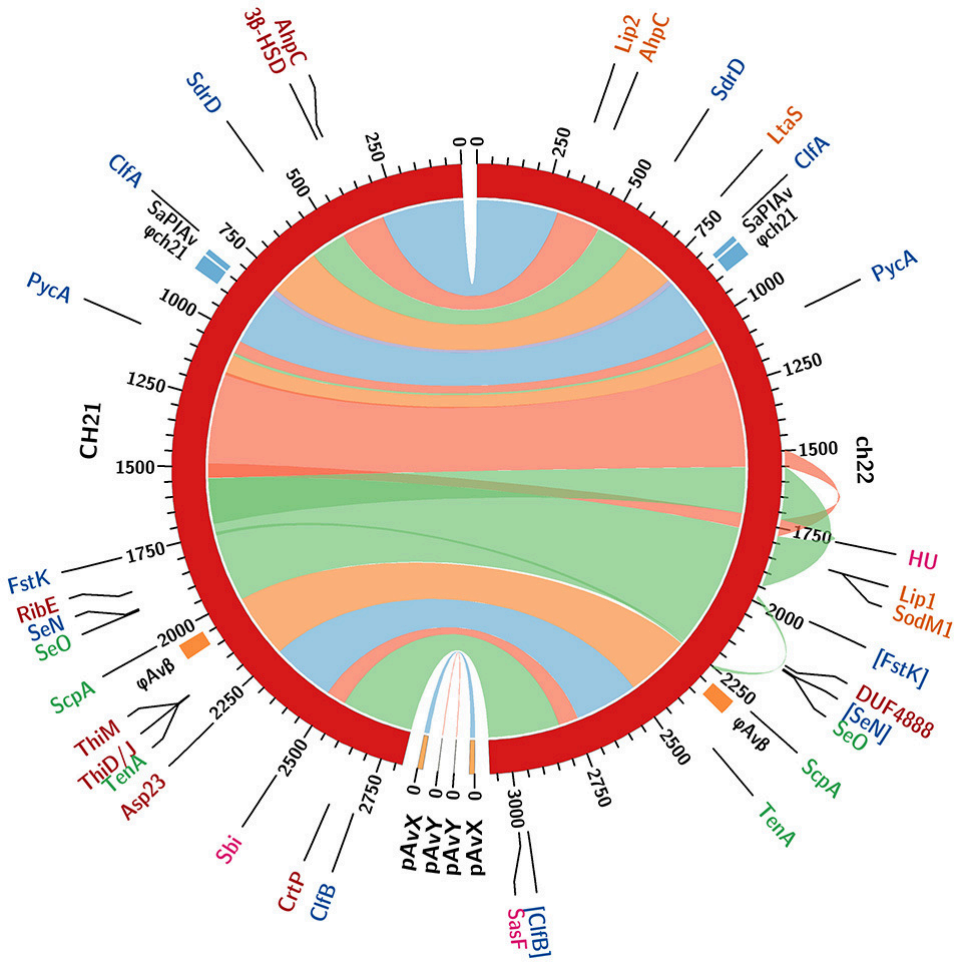
Comparison of whole genome sequences of the CH21 and ch22 strains. Color bands represent continuous similar sequence segments characterizing the two strains. Particular segments are separated by short insertions or duplications unless indicated otherwise. Three longer duplications in the ch22 genome (170, 54, and 11 kbp) are additionally marked by outward ribbons. Loci of intrachromosomal mobile genetic elements, such as prophage φAvβ, putative prophage φch21 and pathogenicity island SaPIAv, are marked. The loci differentiating the two strains are identified with acronyms and colored as follows. Genomics: coding sequences in blue and promoters in green (pseudogenes in square brackets). Proteomics: intracellular in red; extracellular in orange; and surface in magenta (upregulated proteins only).

CH21 and ch22 show opposite phenotypes in a chicken embryo infection model (VIR vs. NVIR, respectively) but are indistinguishable by common typing techniques. We assumed that their different phenotypes may be explained by deep genome sequencing. Such an approach is generally applied to elucidate the genetic bases of the sudden acquisition of antibiotic resistance (Sabat et al., 2015). However, it was likely that the switch between the VIR and NVIR phenotypes in CH21 vs. ch22 was also due to mutations or the loss or acquisition of genetic material, which would be traceable by NGS. Therefore, we obtained the complete genomic sequences of both strains. The first striking difference between the strains was the total length of their genomes, which was 2.8 and 3.1 Mbp, respectively, for the virulent and non-virulent strains. The additional genetic pool of over 250 kbp within the genome of ch22 arose mainly from three duplications. The longest one (CP017807 1552041. . 1722216 and 1777324. . 1947499) encompassed 170 kbp and encoded 230 proteins. Two shorter ones (CP017807 1496929. . 1551606 and 1722212. . 1776889, 54 kbp; and CP017807 1978955. . 1990796 and 2244863. . 2256780, 12 kbp) encoded 70 and 16 proteins, respectively (Supplementary Tables 5–9). Interestingly, the major duplications resided within the core part of the bacterial chromosome, whereas the pool of mobile genetic elements, encompassing plasmids pAvX and pAvY, two prophages and pathogenicity island SaPIAv, remained identical in both strains (Figure 5; Supplementary Table 3). Therefore, the first conclusion is that loss of the virulent phenotype by ch22 was not related to losing a substantial part of its genetic material, which could have been responsible for virulence. On the opposite, pathogenic bacteria were shown to have generally smaller genomes than their close non-virulent relatives (Georgiades and Raoult, 2011). Therefore, one can imagine that the burden of dispensable genetic material could impair the rate of growth and thus the virulence of ch22. However, the growth rate in rich TSB medium as well as in minimal M9-CAA medium were comparable between CH21 and ch22 (Supplementary Figure 6). Moreover, a possible relation between genome size and virulence is not supported by analysis of the genomes of the remaining strains, which have similar sizes within both the VIR and NVIR groups.

A detailed comparative analysis revealed genetic differences that possibly affect the virulence status of CH21 and ch22. The first notable difference between CH21 and ch22 follows the overall distinction characteristic for the analyzed VIR and NVIR strains and encompasses 10 ORFs (Table 2) with established roles in staphylococcal colonization and virulence (Foster et al., 2014). Among others, a deletion and an internal stop codon within the repeat-containing fragments of *clfA* and *clfB* result in the truncation of protein sequences by 113 and 254 amino acid residues, respectively, in CH22. In the case of *sdrD*, eight sense mutations and a point mutation upstream of the coding sequence are different in the strains. CH21 and ch22 are indistinguishable with popular typing methods, including MLVF (Sabat et al., 2003). Significantly, MLVF is based on virulence-related genes and thus allowed us to clearly distinguish virulent (CH3, CH5, CH9, CH21, and CH23) from non-virulent (ch24, pa2, and ph2) strains (Polakowska et al., 2012). This study shows that CH21 and ch22 indeed contain different alleles of three genes (*clfA, clfB*, and *sdrD*) among the seven used for typing; however, the differences were only detectable using deep sequencing. This finding corroborates multiple previous reports on the involvement of these proteins in staphylococcal virulence, where clumping factors and other Sdr proteins facilitate successful colonization-mediating interactions with host proteins, such as fibrinogen, desmoglein-1 and cytokeratin 10 (O'Brien et al., 2002; Walsh et al., 2008; Wertheim et al., 2008; Askarian et al., 2016). As an additional example differentiating CH21 and ch22, the genes encoding pyruvate carboxylase (*pycA*) and exotoxin SeN (*seN*) are pseudogenized in the NVIR ch22 strain (Table 2). Interestingly, *seN* is totally absent in all other NVIR strains. PycA catalyzes the ATP-dependent carboxylation of pyruvate to oxaloacetate, an important anaplerotic reaction that provides intermediates for the tricarboxylic acid cycle (Sauer and Eikmanns, 2005). The activity of PycA requires C-terminal domain-driven tetramerization (Xiang and Tong, 2008). In ch22, an internal stop codon truncates PycA by 110 amino acid residues, most likely disturbing tetramerization and thus protein function. Benton and colleagues (Benton et al., 2004) previously demonstrated the importance of PycA in virulence by showing that the *pycA* mutant was among the most severely attenuated mutants in a murine model of systemic infection. The avirulent phenotype of ch22 is in line with those studies.

**Table 2:** Differences in genes between VIR and NVIR strains exemplified by differences identified in the VIR CH21 and NVIR ch22 strains.

**No**.	Cl.^*^	**Product**	**Acronym**	**CH21 loci**	**ch22 loci**	Protein lengths		**Differences**
1.	188	Hypothetical protein (associated with type VII secretion system operon)		BJL64_01345 BJL64_01350	BJL65_01345 — — — — — —	166 161	161 — —	Merge between two coding sequences of CH21.
2.	438	Serine-aspartate repeat protein D	SdrD	BJL64_02690	BJL65_02680	1,385	1,385	Eight sense mutations, one point mutation upstream of CDS.
3.	670	Clumping factor A	ClfA	BJL64_03875	BJL65_03870	875	762	113 aa deletion within the repeat-containing fragment.
4.	891	Hypothetical protein (associated with bacteriocin operon)		BJL64_05095	[BJL65_05090]	654	— —	Internal stop codon in ch22.
5.	938	Hypothetical protein (lipoprotein)		BJL64_05335	[BJL65_05325]	69	— —	Internal stop codon in ch22.
6.	975	Pyruvate carboxylase	PycA	BJL64_05525	[BJL65_05515]	1,150	— —	Internal stop codon in ch22.
7.	1017	Hypothetical protein		BJL64_05735	[BJL65_05725]	55	— —	Internal stop codon in ch22.
8.	1702	Exotoxin	SeN	BJL64_09410	[BJL65_10575]	251	— —	Internal stop codon in ch22.
9.	1767	Cell division protein	FtsK	BJL64_08675	BJL65_09840	453	453	Five sense mutation and 13 point mutations upstream of CDS.
10.	2529	Clumping factor B	ClfB	BJL64_13955	[BJL65_15235]	865	— —	Internal stop codon in ch22.

* Cluster's number, for detailed protein sequences and alignment see “https://mol058.mol.uj.edu.pl/extra/clusters.htm.”

The second distinction encompasses 12 differences found only between CH21 and ch22, but these differences are not characteristic of the VIR or NVIR groups altogether. In this group, gamma-aminobutyrate permease (*gabP*) is a pseudogene in ch22 but not in CH21, whereas peptide ABC transporter ATP-binding protein (*nikE*) is a pseudogene in CH21 but not in ch22. All remaining differences are sense mutations that affect the amino acid sequence of their respective proteins (Table 3). Interestingly, among the attenuated mutants studied by Benton et al., one mutant carried a mutation in *gabP*, which codes for a transmembrane protein catalyzing the translocation of 4-aminobutyrate [GABA; (Marchler-Bauer et al., 2017)]. In ch22 an internal stop codon truncates the respective protein by 206 amino acid residues. Thus, together with *pycA*, two genes independently demonstrated as important to the virulence of *S. aureus* are switched off in ch22. NikE, pseudogenized in CH21 but not in ch22, is encoded by the *nikBCDE* operon, which constitutes a nickel transport system. Therefore, Ni^2+^ uptake is likely disturbed in CH21, but nickel ions are required for urease activity. An increase in environmental pH due to the production of ammonia by urease was shown to be a significant factor in staphylococcal urinary tract infections (Gatermann and Marre, 1989; Gatermann et al., 1989; Hiron et al., 2010). Moreover, an increase in skin pH due to the activity of arginine deiminase encoded in ACME (Diep et al., 2008) of MRSA USA 300 strains was linked to successful colonization and considered to be a human-host adaptation (Thurlow et al., 2013). One could thus expect that the pseudogenization of *nikE* in CH21 should attenuate virulence, which is however not the case. Instead, we attribute *nikE* pseudogenization to an adaptation to a poultry host. It was shown that the human-to-poultry host jump was associated with the loss of certain genes dispensable for virulence in a new host (such as *spa*, encoding IgG-binding protein A) and the acquisition of other genes (Lowder et al., 2009). Since the pH of chicken skin (pH 6.6–7.2) is significantly higher than that of human skin (pH 5.5), it is reasonable to speculate that an acidic pH is not a significant barrier for poultry colonization by staphylococci. Hence, its increase by urease would be dispensable, and as such, the pseudogenization of *nikE* would not manifest as an attenuation of the virulence of the CH21 strain in a chicken embryo infection model as found in our experiments.

**Table 3:** Identified differences in genes exclusively found between the VIR CH21 and NVIR ch22 strains.

**No**.	Cl.^*^	**Product**	**Acronym**	**CH21 loci**	**ch22 loci**	**Protein lengths**		**Differences**
1.	89	Hypothetical protein		BJL64_00820	BJL65_00820	199	199	Seven sense mutations in a highly variable region.
2.	385	Zinc metalloprotease	FtsH	BJL64_02360	BJL65_02355	710	697	One sense mutation. One SNP resulting in a frameshift at the end of the gene. 36 terminal aa changed to 23 different aa.
3.	475	NAD(P)H-dependent oxidoreductase	WrbA	BJL64_02885	BJL65_02880	178	178	One sense mutation.
4.	1221	Choline transporter	BetT	BJL64_06800	BJL65_06835	548	548	One sense mutation.
5.	1368	Hypothetical protein (lipoprotein)		BJL64_07580	BJL65_07610	230	308	A highly variable region shortened by 78 aa in CH21.
6.	1573	Gamma-aminobutyrate permease	GabP	BJL64_08625	[BJL65_09790]	453	— —	Internal stop codon in ch22.
7.	1700	Hypothetical protein		BJL64_09400	BJL65_10565	258	258	One sense mutation.
8.	1766	AAA family ATPase (FtsK operon associated)	VirB4	[BJL64_08695]	BJL65_09860	— —	832	Internal stop codon in CH21.
9.	2127	Malonate transporter	YfdV	BJL64_11910	BJL65_13180	302	302	Two sense mutations.
10.	2185	Conjugal transfer protein (FtsK operon associated)	TpcC	BJL64_00290 BJL64_08710	BJL65_00290 BJL65_09875	353 358	353 358	Four sense mutations. A variable 17 aa region containing 15 sense mutations.
11.	2356	Peptide ABC transporter ATP-binding protein	NikE	[BJL64_13030]	BJL65_14305	— —	249	Internal stop codon in CH21.
12.	2902	Hypothetical protein		BJL64_11025 — — — — — —	BJL65_12290 BJL65_12295	30 — —	30 30	Identical protein sequences. Tandem duplication. A copy with one point mutation.

* Cluster's number, for detailed protein sequences and alignment see “https://mol058.mol.uj.edu.pl/extra/clusters.htm.”

The last group, differentiating CH21 and ch22, encompasses 19 differences in the promoter regions, which may influence expression of the downstream genes/proteins. The differences range from single nucleotide changes [e.g., fibrinogen-binding protein (FnBP), thiaminase (TenA) and SdrD] to entirely different sequences within the promoter region as exemplified by cysteine protease staphopain A (ScpA; Table 4).

**Table 4:** Identified differences in putative promoter regions between the VIR CH21 and NVIR ch22 strains.

**No**.	**Downstream coding sequence**	**Acronym**	**CH21 loci**	**ch22 loci**	**Protein lengths**		**Differences**
1.	Ring-cleaving dioxygenase	GloA	BJL64_01535	BJL65_01530	308	308	One point mutation upstream of CDS.
2.	Hypothetical protein		BJL64_01915	BJL65_01910	227	227	Completely different sequences from 113 bp upstream of CDS.
3.	Serine-aspartate repeat protein D	SdrD	BJL64_02690	BJL65_02680	1,385	1,385	One point mutation upstream of CDS.
4.	Hypothetical protein		BJL64_04510	BJL65_04505	37	37	Completely different sequences from 145 bp upstream of CDS.
5.	Hypothetical protein		BJL64_05100	BJL65_05095	106	106	Completely different sequences from 54 bp upstream of CDS. In ch22, the preceding CDS is pseudogenized.
6.	Fibrinogen-binding protein	FnBP	BJL64_05765	BJL65_05755	116	116	One single nucleotide deletion upstream of CDS.
7.	Hypothetical protein		BJL64_07020	BJL65_07055	55	55	Five point mutations upstream of CDS.
8.	DNA-binding response regulator	OmpR	BJL64_08605	BJL65_09770	234	234	Three point mutations upstream of CDS.
9.	Hypothetical protein		BJL64_08650	BJL65_09815	120	120	Two point mutations upstream of CDS.
10.	Hypothetical protein (FtsK operon associated)		BJL64_08655	BJL65_09820	197	197	Two point mutations upstream of CDS.
11.	Hypothetical protein (FtsK operon associated)		BJL64_08670	BJL65_09835	77	77	19 point mutations and one single nucleotide deletion upstream of CDS.
12.	Cell division protein	FtsK	BJL64_08675	BJL65_09840	453	453	13 point mutations upstream of CDS.
13.	Hypothetical protein (FtsK operon associated)		BJL64_08680	BJL65_09845	110	110	One point mutation upstream of CDS.
14.	Exotoxin	SeO	BJL64_09435	BJL65_10600	254	254	Four one-nucleotide deletions upstream of CDS.
15.	Cysteine protease (staphopain A)	ScpA	BJL64_10140	BJL65_11405	388	388	Completely different sequences starting from the first bp upstream of CDS because of translocation.
16.	Hypothetical protein		BJL64_11025	BJL65_12290 BJL65_12295	30 30	30	Gene duplication. four point mutations in the region up to 100 bp upstream of CDS and substantial differences in the region from 101 to 200 bp upstream of CDS.
17.	Thiaminase II	TenA	BJL64_11050	BJL65_12320	229	229	One point mutation upstream of CDS.
18.	Hypothetical protein		BJL64_11905	BJL65_13175	43	43	One point mutation upstream of CDS.
19.	Hypothetical protein		BJL64_13105	BJL65_14385	140	140	Deletion of 46 bp right upstream of CDS in CH21.

### Differences in proteomes of CH21 and ch22 strains

**Figure 6:**
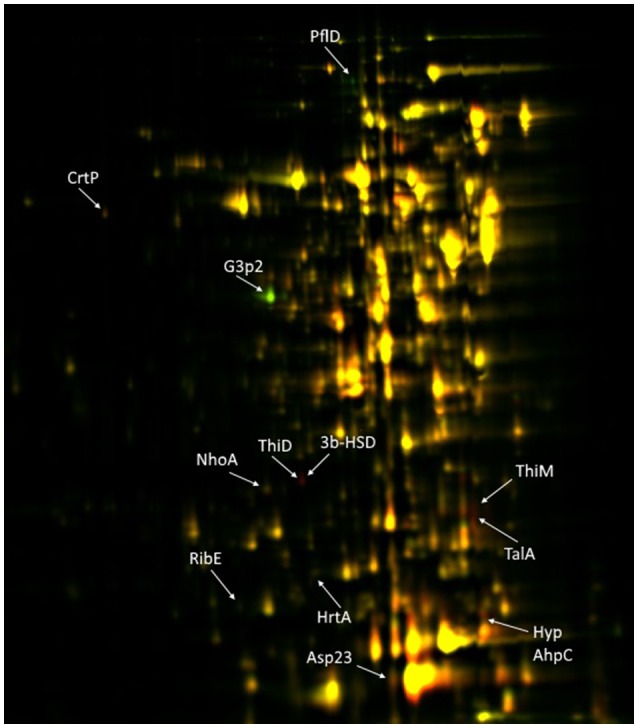
2D-DIGE of cellular proteins isolated from CH21 and ch22 strains. Protein spots positively differentiating in VIR CH21 and NVIR ch22 are more intensive in red and green canal, respectively. Protein spots with equal expression in both strains are yellow. Identified differentiating proteins are marked with acronyms.

Genomic comparison of the CH21 and ch22 strains provided certain clues regarding the likely genetic determinants of differences in the virulent phenotype. Nevertheless, the influence of genetic alterations on the overall proteomic spectrum remains hard to reliably predict and thus was tested experimentally. We have previously compared the exoproteomes of a number of VIR and NVIR *S. aureus* strains to demonstrate the extracellular fingerprints of each phenotype (Bonar et al., 2016). Here, we focused on comparing the cellular, cell wall/membrane associated and secreted proteomes of two genetically related strains CH21 and ch22 to pinpoint the characteristics determining their different virulence phenotypes. Among the cellular proteins, 12 differentiating spots were identified as characteristic of CH21 compared to ch22, whereas eight spots were characteristic of ch22. MS analysis identified 10 and three unique proteins in the respective groups of differentiating spots (Table 5; Figure 6; Supplementary Table 13).

**Table 5:** List of proteins differentially expressed by VIR CH21 and NVIR ch22 strains as identified by proteomics.

**Location**	**Locustag CH21**	**Locustag ch22**	**Protein**	**Acronym**	**Claster of orthologous groups (functional category)**	**Elevated in/shaved from strain**
in	BJL64_13565	BJL65_14845	Diapolycopene oxygenase	CrtP	1233 (Q)	CH21
in	BJL64_11045	BJL65_12315	Bifunctionalhydroxymethylpyrimidine kinase/phosphomethylpyrimidine kinase	ThiD/J	0351 (H)	CH21
in	BJL64_01840	BJL65_01835	NAD(P)-dependent oxidoreductase	3β-HSD	0702 (R)	CH21
in	BJL64_14305	BJL65_15585	Arylamine N-acetyltransferase	NhoA	2162 (Q)	CH21
in	BJL64_11040	BJL65_12310	Hydroxyethylthiazole kinase	ThiM	2145 (H)	CH21
in	BJL64_09170	BJL65_10335	Translaldolase	TalA	0176 (G)	CH21
in	BJL64_09115	BJL65_10280	Riboflavin synthase subunit alpha	RibE	0307 (H)	CH21
in	BJL64_00690	BJL65_00690	Hypothetical protein	Hyp	n/a	CH21
in	BJL64_01760	BJL65_01755	Peroxiredoxin	AhpC	0450 (V)	CH21
in	BJL64_11525	BJL65_12795	Alkalineshock protein	Asp23	1302 (S)	CH21
in	BJL64_00965	BJL65_00965	Formate C-acetyltransferase	PflD	1882 (C)	ch22
in	BJL64_08575	BJL65_09740	Type I glyceraldehyde-3-phosphate dehydrogenase	G3p2	0057 (G)	ch22
in	BJL64_12445	BJL65_13715	Hemin ABC transporter ATP-binding protein	HrtA	1136 (M)	ch22
out	BJL64_09350	BJL65_10515	DUF4888 domain-containing protein	DUF4888	n/a	CH21
out	BJL64_02695	BJL65_02685	MSCRAMM family adhesion SdrE	SdrE	4932 (S)	ch22
out	BJL64_07905	BJL65_09090	Superoxide dismutase	SodM1	0605 (P)	ch22
out	BJL64_01760	BJL65_01755	Peroxiredoxin	AhpC	0450 (V)	ch22
out	BJL64_03485	BJL65_03480	Glycerol phosphate lipoteichoic acid synthase	LtaS	1368 (M)	ch22
out	BJL64_01445	BJL65_01440	Lipase2	Lip2	1075 (I)	ch22
out	BJL64_11645	BJL65_12915	Toxin		n/a	ch22
out	BJL64_14165	BJL65_15445	Lipase1	Lip1	1075 (I)	ch22
surf	BJL64_02580	BJL65_02570	50S ribosomal protein L7/L12	RL7/12	0222 (J)	CH21
surf	BJL64_12760	BJL65_14035	Immunoglobulin-binding protein Sbi	Sbi	n/a	CH21
surf	BJL64_14035	BJL65_15315	N-acetylmuramoyl-L-alanine amidase	Y2979	1705 (MN)	CH21
surf	BJL64_03805	BJL65_03800	Phosphopyruvate hydratase	Eno	0148 (G)	ch22
surf	BJL64_07465	BJL65_08690	DNA-binding protein	HU	0776 (L)	ch22
surf	BJL64_11780	BJL65_13050	30S ribosomal protein S5	RS5	0098 (J)	ch22
surf	BJL64_13785	BJL65_15065	Fructose bisphosphate aldolase	Alf2	3588 (G)	ch22
surf	BJL64_14045	BJL65_15325	Adhesin (surface protein F)	SasF	n/a	ch22

Although intracellular proteins do not directly interact with the host, they significantly contribute toward maintaining the virulent phenotype by maintaining metabolism in stress conditions. Proteins characterized by more abundant expression in virulent strain CH21 compared to ch22 included diapolycopene oxygenase (CrtP), which is involved in the biosynthesis of staphyloxanthin. Interestingly, however both strains exhibited the same level of pigmentation (Supplementary Figure 5). This golden pigment has been linked with staphylococcal virulence as it shields the microbe from oxidation-based clearance, which is the innate host immune response to infection (Clauditz et al., 2006). Interestingly, it has been shown that strains with gene knock-outs in the staphyloxanthin biosynthesis pathway exhibit attenuated virulence (Liu et al., 2005). Nevertheless, staphyloxanthin overexpression does not directly correlate with virulence. It has been shown that strains with elevated pigmentation (associated with mutations in genes responsible for oxidative phosphorylation and purine biosynthesis) were less virulent in a murine abscess model of infection. Some strains were even unable to exhibit long-term colonization (Lan et al., 2010). Furthermore, supplementation of *S. argenteus* with a gene cluster responsible for staphyloxanthin production led to increased susceptibility to the host defense peptides LL-37 and hNP-1 *in vitro* and reduced virulence in an experimental rabbit endocarditis model (Xiong et al., 2015). Therefore, CrtP overexpression in CH21 may not necessarily relate to the virulence of this strain.

Peroxiredoxin, another enzyme protecting these bacteria against oxidative host attack by scavenging hydrogen peroxide, was also upregulated in CH21 compared to ch22. This finding demonstrates the general importance of antioxidative mechanisms in staphylococcal virulence. The level of alkaline shock protein (Asp23), which was linked with cell homeostasis and protection of the cell envelope in non-growing cells (Müller et al., 2014), was also elevated in CH21 compared to ch22. In addition, enzymes implicated in the synthesis of vitamins [thiamine (hydroxymethylpyrimidine/phosphomethylpyrimidine kinase and hyroxyethylthiazole kinase), and riboflavin (riboflavin synthase subunit alpha)] and an enzyme involved in the metabolism of bile acid [NAD(P)-dependent oxidoreductase] were also elevated in the virulent CH21 strain compared to ch22; however, the significance of this fact in virulence remains elusive.

Proteins characterized by higher expression in non-virulent strain ch22 compared to virulent strain CH21 included type I glyceraldehyde-3-phosphate dehydrogenase 2 (G3P2), an isoenzyme of G3PD-1 involved in glycolysis, and formate C-acetyltransferase, which is implicated in anaerobic glucose metabolism. Hemin ABC transporter ATP-binding protein (HrtA) was also elevated in ch22 (Figure 6). HrtA is responsible for coping with heme stress and was already demonstrated to negatively correlate with staphylococcal virulence (Hammer and Skaar, 2011). The transporter prevents heme-mediated toxicity. It has been demonstrated that an inability to cope with heme stress paradoxically yields a hypervirulent phenotype (Hammer and Skaar, 2011). In *hrtA* mutants, such a phenotype was associated with the loss of membrane integrity and increased secretion of immunomodulatory proteins (Attia et al., 2010). The above findings point to the likely significance of the inability to produce HrtA by CH21 in maintaining the strain virulence.

In conclusion, although the expression of only 13 intracellular proteins is significantly different between the VIR and NVIR strains, the pattern of expression is strikingly consistent. VIR strain CH21 over-expresses proteins involved in coping with oxidative stress, which characterizes the host response against invading pathogens. In contrast, NVIR strain ch22 overproduces enzymes adapting the cell to anaerobic conditions, which characterize its niches of commensal coexistence with the host.

The exoproteomes of strains CH21 and ch22 are dominated by staphopain C, a plasmid-encoded cysteine protease overproduced by a range of poultry-derived *S. aureus* strains that was previously demonstrated to bea factor unrelated to virulence (Bonar et al., 2016). Only three differentiating spots were identified as overexpressed within the exoproteome of virulent strain CH21 compared to ch22, and all three spots contained DUF4888 domain-containing protein (Table 5; Supplementary Table 13). This protein of 193 residues has no known function (Marchler-Bauer et al., 2017). Homologes are primarily found in various species of staphylococci, suggesting a species-specific role, the elucidation of which is of significant interest in the light of this study. Interestingly, the exoproteome of the virulent strain CH21 is not characterized by the overexpression of any known virulence factors compared to nonvirulent strain ch22. Even alpha-hemolysin, which was previously consistently found to be overexpressed in the exoproteomes of virulent strains isolated from poultry, is not overproduced by CH21 compared to ch22 (Bonar et al., 2016).

Within the exoproteome of NVIR strain ch22, seven proteins were found to be overexpressed compared to CH21 (Table 5; Supplementary Table 13). The most pronounced differences in expression were characterized by lipase 1 (Lip1, found in 15 differentiating protein spots) and lipoteichoic acid synthase (LtaS, found in 10 differentiating spots). Further, peroxiredoxin (AhpC) was identified in three differentiating spots, whereas superoxide dismutase 1 (SodM1) and lipase 2 (Lip2) were found in two differentiating spots. Additional proteins were identified as single differentiating spots.

**Figure 7:**
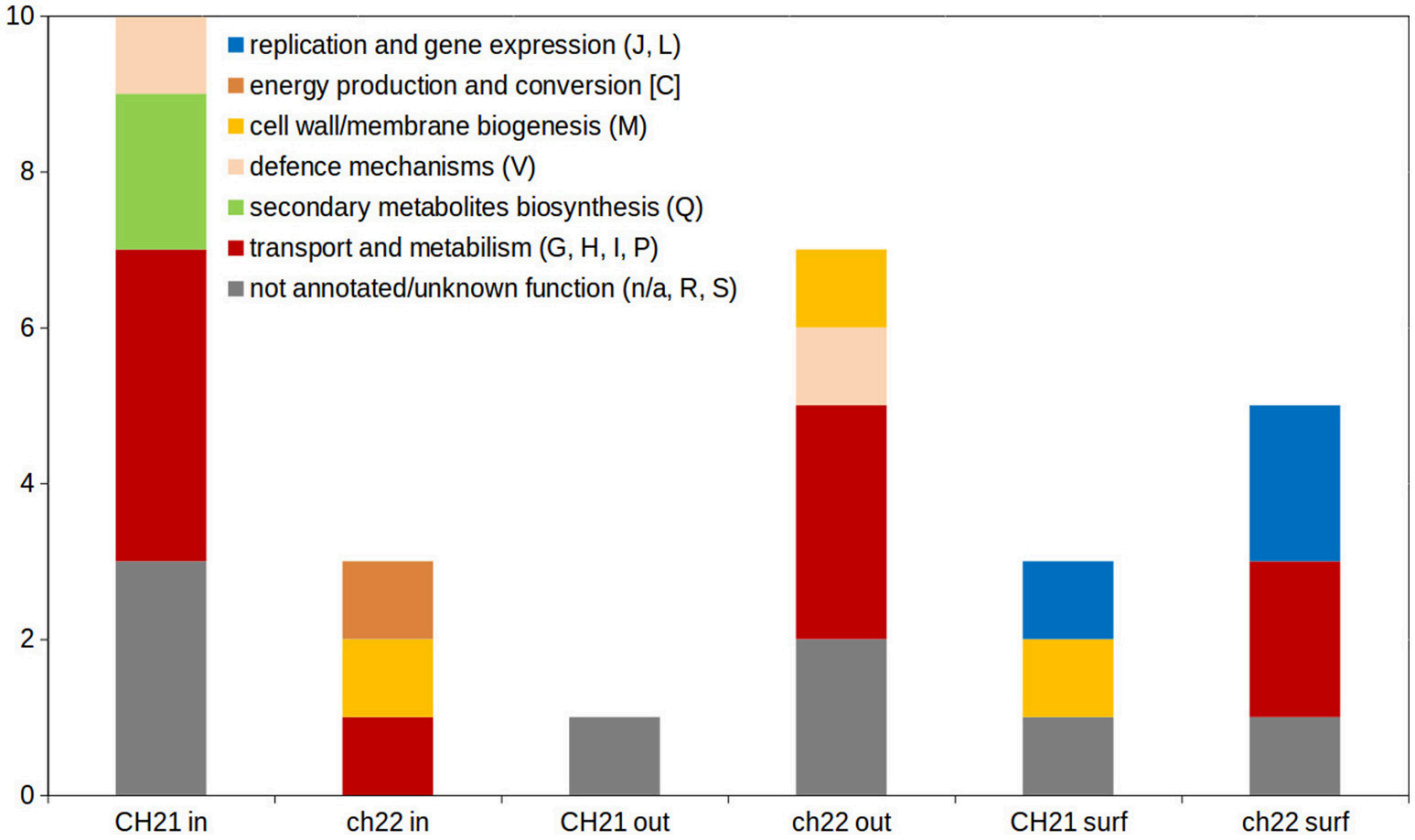
Distribution of functional categories of Clusters of Orthologous Groups (COGs) within differentiating proteins identified during analysis of intracellular (in), extracellular (out) and cell membrane/wall-associated (surf) proteome. One letter code of functional categories corresponds to the code in COGs database [https://www.ncbi.nlm.nih.gov/COG/; (Galperin et al., 2015)].

The higher complexity of the exoproteome of nonvirulent strain ch22 compared to virulent strain CH21 corroborates the results of our previous study, which documented a general regularity in the more complexed exoproteomes of NVIR strains relative to VIR strains (Bonar et al., 2016). Moreover, Clusters of Orthologous Groups (COGs) analysis confirmed that proteins differentiating both exo- and intracellular proteomes of CH21 and ch22 were assigned to different functional categories (Figure 7; Supplementary Figure 4).

Gel proteomics performs poorly in comparing cell membrane / cell wall associated proteomes. Therefore, to compare these subsets of the proteomes of CH21 and ch22, we used the cell “shaving” approach (Solis et al., 2010) coupled to direct, LC-MS based identification. This approach enabled direct comparisons, but unlike semiquantitative gel proteomics, the direct approach is qualitative only and suffers from large variability among pools identified in different biological repeats. As such, only identifications present in two or more samples were considered further (Table 5; Supplementary Table 13). Proteins identified exclusively on the surface of virulent strain CH21 include immunoglobulin-binding protein (Sbi) and N-acetylmuramoyl-L-alanine amidase (Y2979; BJL64_14035). Sbi and staphylococcal protein A (SpA) are IgG binders with a demonstrated role in staphylococcal virulence (Gonzalez et al., 2015). While SpA binds Fcγ of IgGs, Sbi has two Ig-binding domains and two domains that bind to complement component C3 (Zhao et al., 2016). A prior study demonstrated the pseudogenization of *spa*, a gene encoding a major *S. aureus* IgG-binding protein, as a specific adaptation to a poultry host since SpA is unable to bind chicken immunoglobulins (Lowder et al., 2009). It is tempting to speculate that Sbi may replace SpA in poultry strains. Sbi has a broader ability to bind mammalian IgG than SpA, but its ability to bind avian Igs remains to be tested (Atkins et al., 2008). Amidase Y2979 is consistently found in the exoproteomes of different virulent *S. aureus* strains isolated from poultry (Bonar et al., 2016), but its role in staphylococcal virulence remains unknown.

SasF adhesin and DNA-binding protein (HU) are distinguished among proteins identified exclusively on the surface of non-virulent strain ch22. SasF was initially identified as a cell wall attached adhesion protein (Roche et al., 2003), but later, it was demonstrated that it provides resistance to unsaturated free fatty acids, such as linoleic acid (Kenny et al., 2009). It is thus likely that SasF has a protective role, compensating the increased lipase content in the exoproteome of ch22. High lipase would result in high production of free fatty acids, which in turn, could adversely affect the integrity of the bacterial cell membrane (Cadieux et al., 2014). SasF would counteract such adverse effects. HU protein is one of the major nucleoid-associated proteins involved in DNA bending and thus the determination of prokaryotic chromosome structure (Kim et al., 2014a). It also acts as a transcriptional regulator of genes, responding to anaerobiosis, acid stress, high osmolarity and SOS induction (Oberto et al., 2009). Abundant intracellular proteins are often concomitantly present at the surface of these bacteria, and they usually have moonlighting functions. Extracellular DNA is an important component of bacterial biofilms, and DNA-binding proteins other than HU are associated with biofilm formation (Joo and Otto, 2012), suggesting that HU may have a similar function.

### Advantageous influence of “individualized” genomics in proteomic identifications

The number of studies combining genomic and proteomic approaches is relatively limited, but the complexity of host-pathogen interactions calls for such a holistic approach. By applying the advantage of the growing availability of NGS, this study, for the first time, based proteomic identifications on “individualized” information retrieved from the genomes of the studied strains. More importantly, however, such an approach enabled the correlation of genomic and proteomic data, providing rational explanations for the varied expression of specific proteins differentiating the virulent and nonvirulent strains. Bifunctional hydroxymethylpyrimidine kinase/phosphomethylpyrimidine kinase (BJL64_11045) and hydroxyethylthiazole kinase (BJL64_11040), proteins upregulated in CH21 compared to ch22, are encoded in a single operon (Müller et al., 2009) together with the thiaminase gene (BJL64_11050, TenA). Genomics demonstrates a single nucleotide polymorphism in the putative promoter region of the operon in which a polymorphism may be responsible for differential expression. Even more interestingly, in the case of superoxide dismutase (BJL65_09090) and DNA-binding protein (BJL65_08690; HU), which were upregulated in ch22 compared to CH21, additional copies (BJL65_07935 and BJL65_07500, respectively) of the encoding genes were found in 170 and 54 kbp duplications, respectively, explaining the observed increased expression. Nevertheless, of the 28 proteins differentially expressed in CH21 and ch22, differences in the expression of only the above four proteins could have been explained on genetic basis. This result demonstrates that global expression regulators influence the proteome more significantly than alterations in particular promoter regions. Superoxide dismutases (SODs) give bacteria a defense mechanism against professional phagocytes. Mn-dependent SOD (SodM1) is specific for *S. aureus* and not found in coagulase-negative staphylococci, a group of staphylococci that is generally less pathogenic than the former species (Wright Valderas et al., 2002). SodM1 is generally a cytosolic protein, however extracellular SodM1 was previously identified and implicated in biofilm formation (Atshan et al., 2015). Although CH21 and ch22 are weak biofilm producers, the latter indeed forms a slightly higher biofilm when tested *in vitro* than the former (Supplementary Figure 3). In the above context, it is interesting to note that another primarily intracellular DNA-binding protein (HU), identified in the exoproteome of ch22, may be involved in biofilm formation. Extracellular DNA is an important component of bacterial biofilms, and DNA-binding proteins have been associated with biofilm formation (Joo and Otto, 2012). The importance of such moonlighting proteins is shown by the fact that specific monoclonal antibodies are able to disrupt an established biofilm and restore the antibiotic sensitivity of released bacteria (Estellés et al., 2016). Nonetheless, it remains to be determined whether the overexpression of HU by ch22 truly influences biofilm formation and virulence.

In conclusion, staphylococcal virulence has been investigated using genetic, proteomic, biochemical and molecular approaches (Hecker et al., 2018). It is currently well-established that the virulent phenotype relies on multiple factors that are subject to fine-tuned regulation and act in a highly orchestrated manner (Thomer et al., 2016). Nevertheless, the corresponding interconnections and guiding principles remain elusive. Here, we demonstrated that the level of virulence of wild-type *S. aureus* strains may be significantly influenced by minor differences in their genetic material. Performed proteomics indicate that coping with oxidative stress is crucial for virulent strain CH21, whereas basic metabolism and nutrient acquisition are fine-tuned in non-virulent ch22. However, we are also aware that the study was performed on a limited number of strains and changes in proteomes were assessed using *in vitro* bacterial cultures which not optimally reflects *in vivo* conditions. Nevertheless, we pointed mutations in wild-type *S. aureus* which have been previously showed to be linked with staphylococcal virulence using recombinant strains (Benton et al., 2004), which strengthen accuracy of our findings. Other results are candidates for further detailed studies.

## Author contributions

EB, MB, and BW designed the study. EB, MB, MH, UJ, SK-K, VA, and AS performed the experiments. EB, MB, SK-K, MG, GD, JM, AS, AF, and BW analyzed and interpreted data. EB, MB, GD, and BW wrote the manuscript. All authors revised the manuscript and agreed to be accountable for all aspects of the work herein.

### Conflict of interest statement

The authors declare that the research was conducted in the absence of any commercial or financial relationships that could be construed as a potential conflict of interest.
