# Supplementary material for: Joint Genomic and Proteomic Analysis Identifies Meta-Trait Characteristics of Virulent and Non-virulent Staphylococcus aureus Strains
Source: Front Cell Infect Microbiol. 2018 Sep 6;8:313. doi: 10.3389/fcimb.2018.00313 (PMC6136393; doi:10.3389/fcimb.2018.00313)
Supplement: Supplementary file 1 [file Data_Sheet_1.PDF]

Joint Genomic and Proteomic Analysis Identifies Meta-trait Characteristics of Virulent and Non-virulent *Staphylococcus aureus* Strains

Emilia Bonar[1,#], Michal Bukowski[1,#], Marcin Hydzik[1], Urszula Jankowska[2], Sylwia Kedracka-Krok[3], Magdalena Groborz[1], Grzegorz Dubin[2,4], Viktoria Akkerboom[5], Jacek Miedzobrodzki[4], Artur J. Sabat[5], Alexander W. Friedrich[5], Benedykt Wladyka[1,*]

[1]Department of Analytical Biochemistry, Faculty of Biochemistry, Biophysics and Biotechnology, Jagiellonian University, Krakow, Poland
[2]Malopolska Centre of Biotechnology, Jagiellonian University, Krakow, Poland
[3]Department of Physical Biochemistry, Faculty of Biochemistry, Biophysics and Biotechnology, Jagiellonian University, Krakow, Poland
[4]Department of Microbiology, Faculty of Biochemistry, Biophysics and Biotechnology, Jagiellonian University, Krakow, Poland
[5]Department of Medical Microbiology, University Medical Center Groningen, University of Groningen, Groningen, Netherlands

[#] equal contribution
[*] corresponding author
Dr. Benedykt Wladyka
E-mail: benedykt.wladyka@uj.edu.pl

Contents

Supplementary table 1 ………………………….…. 2
Supplementary table 2 ………………….…….….… 3
Supplementary table 3 …………………………….. 8
Supplementary table 4 …………………………….. 9
Supplementary table 5 …………………………… 10
Supplementary table 6 …………………………… 15
Supplementary table 7 …………………………… 17
Supplementary table 8 …………………………… 18
Supplementary table 9 …………………………… 18
Supplementary table 10 …………………………… 19
Supplementary table 11 …………………………… 22
Supplementary table 12 …………………………… 29
Supplementary table 13 …………………………… 30

Supplementary figure 1 ……………………….…. 32
Supplementary figure 2 …………………………… 32
Supplementary figure 3 …………………………… 33
Supplementary figure 4 …………………………… 34
Supplementary figure 5 …………………………… 35
Supplementary figure 6 …………………………… 36

Suppl. tab. 1. Database sequence records associated with the analysed strains (BioProject ID: PRJNA344860).

Strain	Status	Accession numbers	Contigs	Total length	Coding sequences
ch3	WGS	MOYG00000000	55	2 798 548	2 800
ch5	WGS	MSGQ00000000	61	2 834 199	2 919
ch9	WGS	MOYH00000000	45	2 794 799	2 803
ch21	complete	CP017804, CP017805, CP017806	3	2 841 298	2 871
ch22	complete	CP017807, CP017808, CP017809	3	3 092 777	3 073
ch23	WGS	MOYI00000000	50	2 809 930	2 820
ch24	WGS	MOYJ00000000	60	2 861 584	2 875
pa3	WGS	MOXP00000000	85	2 804 495	2 822
ph2	WGS	MOYK00000000	73	2 815 966	2 828

Suppl. tab. 2. Database sequence records of mobile genetic elements (MGEs) used for prophages and pathogenicity islands identification.

Phages

**No.**	**Definition**	**Accession**
1.	*Staphylococcus* phage PVL proviral DNA, complete sequence	AB009866
2.	*Staphylococcus* phage phiSLT DNA, complete genome	AB045978
3.	*Staphylococcus* phage phiPVL108 DNA, complete genome	AB243556
4.	*Staphylococcus* phage phiMR25 DNA, complete genome	AB370205
5.	*Staphylococcus* phage phiMR11 DNA, complete genome	AB370268
6.	*Staphylococcus* phage S24-1 DNA, complete genome	AB626962
7.	*Staphylococcus* phage S13' DNA, complete genome	AB626963
8.	*Staphylococcus* phage S25-3 DNA, complete genome	AB853330
9.	*Staphylococcus* phage S25-4 DNA, complete genome	AB853331
10.	*Staphylococcus* phage phiSA012 DNA, complete genome	AB903967
11.	*S. aureus* phage phi 11, complete genome	AF424781
12.	*S. aureus* phage phi 12, complete genome	AF424782
13.	*S. aureus* phage phi 13, complete genome	AF424783
14.	*Staphylococcus* phage phi44AHJD, complete genome	AF513032
15.	*S. aureus* phage phiP68, complete genome	AF513033
16.	*Staphylococcus* phage phiETA DNA, complete genome	AP001553
17.	*Staphylococcus* phage phiETA2 DNA, complete genome, strain: TY94	AP008953
18.	*Staphylococcus* phage phiETA3 DNA, complete genome, strain: TY32	AP008954
19.	*Staphylococcus* phage phi5967PVL DNA, complete genome	AP011955
20.	*Staphylococcus* phage phi7247PVL DNA, complete genome	AP011956
21.	*Staphylococcus* phage phi7401PVL DNA, complete genome	AP012341
22.	*Staphylococcus* phage Twort, complete genome	AY954970
23.	*Staphylococcus* phage 80alpha, complete sequence	DQ517338
24.	*S. aureus* phage phiNM1, complete genome	DQ530359
25.	*S. aureus* phage phiNM2, complete genome	DQ530360
26.	*S. aureus* phage phiNM3, complete genome	DQ530361
27.	*S. aureus* phage phiNM4, complete genome	DQ530362
28.	*Staphylococcus* phage CNPH82, complete genome	DQ831957
29.	*Staphylococcus* phage PH15, complete genome	DQ834250
30.	*Staphylococcus* phage 80, complete genome	DQ908929
31.	*Staphylococcus* phage tp310-1, complete sequence	EF462197
32.	*Staphylococcus* phage tp310-2, complete sequence	EF462198
33.	*Staphylococcus* phage tp310-3, complete sequence	EF462199
34.	*Staphylococcus* phage SAP-2, complete genome	EU136189
35.	*Staphylococcus* phage A5W, complete genome	EU418428
36.	*Staphylococcus* phage phiSauS-IPLA88, complete genome	EU861004
37.	*Staphylococcus* phage phiSauS-IPLA35, complete genome	EU861005
38.	*Staphylococcus* phage phiPVL-CN125, complete genome	FJ713816
39.	*Staphylococcus* phage ISP complete genome	FR852584
40.	*Staphylococcus* phage P954, complete genome	GQ398772

41. *Staphylococcus* phage SA1, complete genome	GU169904
42. *Staphylococcus* phage SAP-26, complete genome	GU477322
43. *Staphylococcus* phage SpaA1 complete genome	HE614281
44. *Staphylococcus* phage PSa3 complete sequence	HF937074
45. *Staphylococcus* phage TEM126, complete sequence	HQ127381
46. *Staphylococcus* phage Sb-1, complete genome	HQ163896
47. *Staphylococcus* phage vB_SepiS-phiIPLA5, complete genome	JN192400
48. *Staphylococcus* phage vB_SepiS-phiIPLA7, complete genome	JN192401
49. *Staphylococcus* phage StB27, complete genome	JN700519
50. *Staphylococcus* phage StB12, complete genome	JN700520
51. *Staphylococcus* phage StB20, complete genome	JN700521
52. *Staphylococcus* phage GH15, complete genome	JQ686190
53. *Staphylococcus* phage SMSAP5, complete genome	JQ779023
54. *Staphylococcus* phage TEM123, complete genome	JQ779024
55. *Staphylococcus* phage StauST398-2, complete genome	JQ957932
56. *Staphylococcus* phage StauST398-3, complete genome	JQ973847
57. *Staphylococcus* phage StauST398-1, complete genome	JX013863
58. *Staphylococcus* phage Staph1N, complete genome	JX080300
59. *Staphylococcus* phage A3R, complete genome	JX080301
60. *Staphylococcus* phage 676Z, complete genome	JX080302
61. *Staphylococcus* phage Fi200W, complete genome	JX080303
62. *Staphylococcus* phage MSA6, complete genome	JX080304
63. *Staphylococcus* phage P4W, complete genome	JX080305
64. *Staphylococcus* phage SA13, complete genome	JX094501
65. *Staphylococcus* phage LH1, complete genome	JX174275
66. *Staphylococcus* phage SA11, complete genome	JX194239
67. *Staphylococcus* phage SP5, complete genome	JX274646
68. *Staphylococcus* phage SP6, complete genome	JX274647
69. *Staphylococcus* phage vB_SauM_Remus, complete genome	JX846612
70. *Staphylococcus* phage vB_SauM_Romulus, complete genome	JX846613
71. *Staphylococcus* phage SA5, complete genome	JX875065
72. *Staphylococcus* phage JD007, complete genome	JX878671
73. *Staphylococcus* phage Team1, complete genome	KC012913
74. *Staphylococcus* phage JS01, complete genome	KC342645
75. *Staphylococcus* phage StauST398-4, complete genome	KC595278
76. *Staphylococcus* phage StauST398-5, complete genome	KC595279
77. *Staphylococcus* phage SA12, complete genome	KC677663
78. *Staphylococcus* phage phiIBB-SEP1, complete genome	KF021268
79. *Staphylococcus* phage phiRS7, complete genome	KF589919
80. *Staphylococcus* phage YMC/09/04/R1988, complete genome	KF598856
81. *Staphylococcus* phage K, complete genome	KF766114
82. *Staphylococcus* phage phiBU01, complete genome	KF831354
83. *Staphylococcus* phage vB_SepS_SEP9, complete genome	KF929199
84. *Staphylococcus* phage DW2, complete genome	KJ140076
85. *Staphylococcus* phage 812, complete genome	KJ206559

86. *Staphylococcus* phage GRCS, complete genome	KJ210330
87. *Staphylococcus* phage 3MRA, complete genome	KJ452291
88. *Staphylococcus* phage 23MRA, complete genome	KJ452292
89. *Staphylococcus* phage phiSa119, complete genome	KJ596420
90. *Staphylococcus* phage SA97, complete genome	KJ716334
91. *Staphylococcus* phage 6ec, complete genome	KJ804259
92. *Staphylococcus* phage MCE-2014, complete genome	KJ888149
93. *Staphylococcus* phage P108, complete genome	KM216423
94. *Staphylococcus* phage BP39, complete genome	KM366100
95. *Staphylococcus* phage phiIPLA-RODI, complete genome	KP027446
96. *Staphylococcus* phage phiIPLA-C1C, complete genome	KP027447
97. UNVERIFIED: *Staphylococcus* phage PhiNM4-gamma4, complete genome	KP209285
98. *Staphylococcus* phage IME-SA1, complete genome	KP687431
99. *Staphylococcus* phage IME-SA2, complete genome	KP687432
100. *Staphylococcus* phage IME-SA4, complete genome	KP735928
101. *Staphylococcus* phage Stau2, complete genome	KP881332
102. *Staphylococcus* phage B166, complete genome	KP893289
103. *Staphylococcus* phage B236, complete genome	KP893290
104. *Staphylococcus* phage 55-2, complete genome	KR709302
105. *Staphylococcus* phage 55-3, complete genome	KR709303
106. *Staphylococcus* phage IME-SA118, complete genome	KR902361
107. *Staphylococcus* phage IME-SA119, complete genome	KR908644
108. *Staphylococcus* phage vB_SauS_phi2, complete genome	KT186243
109. *Staphylococcus* phage phiJB, complete genome	KT344895
110. *Staphylococcus* phage SPbeta-like, complete genome	KT429160
111. *Staphylococcus* phage StB20-like, complete genome	KT429161
112. *Staphylococcus* phage CNPx, complete genome	KU598975
113. *Staphylococcus* phage SLPW, complete genome	KU992911
114. *Staphylococcus* phage pSco-10, complete genome	KX011028
115. *Staphylococcus* phage PVL-Sa2GN1 proviral DNA, lukS-PV, lukF-PV genes, insertion sequence:IS1272, complete cds and complete sequence	LC086374
116. *S. aureus* DNA, phage Sa3 (OC8), complete sequence	LC129040
117. *Staphylococcus* phage 77, complete genome	NC_005356
118. *Staphylococcus* phage PT1028, complete genome	NC_007045
119. *Staphylococcus* phage 66, complete genome	NC_007046
120. *Staphylococcus* phage 187, complete genome	NC_007047
121. *Staphylococcus* phage 69, complete genome	NC_007048
122. *Staphylococcus* phage 53, complete genome	NC_007049
123. *Staphylococcus* phage 85, complete genome	NC_007050
124. *Staphylococcus* phage 2638A, complete genome	NC_007051
125. *Staphylococcus* phage 42e, complete genome	NC_007052
126. *Staphylococcus* phage 3A, complete genome	NC_007053
127. *Staphylococcus* phage 47, complete genome	NC_007054
128. *Staphylococcus* phage 37, complete genome	NC_007055
129. *Staphylococcus* phage EW, complete genome	NC_007056

130.	*Staphylococcus* phage 96, complete genome	NC_007057
131.	*Staphylococcus* phage ROSA, complete genome	NC_007058
132.	*Staphylococcus* phage 71, complete genome	NC_007059
133.	*Staphylococcus* phage 55, complete genome	NC_007060
134.	*Staphylococcus* phage 29, complete genome	NC_007061
135.	*Staphylococcus* phage 52A, complete genome	NC_007062
136.	*Staphylococcus* phage 88, complete genome	NC_007063
137.	*Staphylococcus* phage 92, complete genome	NC_007064
138.	*Staphylococcus* phage X2, complete genome	NC_007065
139.	*Staphylococcus* phage G1, complete genome	NC_007066
140.	*Staphylococcus* phage phi2958PVL, complete genome	NC_011344
141.	*S. aureus* ED98, phage φAv1, complete genome	NC_013450
142.	*S. aureus* ED98, phage φAvβ, complete genome	NC_013450

Pathogenicity islands

No.	Definition	Accession
1.	*S. aureus* DNA, pathogenicity island SaPIj50, complete sequence, strain: NN50	AB679717
2.	*S. aureus* DNA, pathogenicity island, complete sequence, strain: NN54	AB690437
3.	*S. aureus* DNA, pathogenicity island, complete sequence, strain: PM1	AB690438
4.	*S. aureus* DNA, pathogenicity island SaPIivm60, complete sequence, strain: IVM60	AB704539
5.	*S. aureus* DNA, pathogenicity island SaPIhhms2, complete sequence, strain: HHMS2	AB704540
6.	*S. aureus* DNA, pathogenicity island SaPIj11, complete sequence, strain: J11	AB704541
7.	*S. aureus* DNA, pathogenicity island, strain: IVM10	AB716349
8.	*S. aureus* DNA, pathogenicity island, strain: Ishikawa11	AB716350
9.	*S. aureus* DNA, pathogenicity island, strain: No. 10	AB716351
10.	*S. aureus* DNA, pathogenicity island, strain: Hirosaki4	AB716352
11.	S. *epidermidis* DNA, pathogenicity island region, strain: NTUH-3692	AB828059
12.	S. *epidermidis* DNA, pathogenicity island region, strain: NTUH-857	AB828060
13.	*S. aureus* DNA, pathogenicity island SaPITokyo12413, complete sequence, strain: Tokyo12413	AB860415
14.	*S. aureus* DNA, pathogenicity island SaPITokyo11212, complete sequence, strain: Tokyo11212	AB860416
15.	*S. aureus* DNA, pathogenicity island SaPITokyo12571, complete sequence, strain: Tokyo12571	AB860417
16.	*S. aureus* DNA, pathogenicity island SaPITokyo12381, complete sequence, strain: Tokyo12381	AB860418
17.	*S. aureus* DNA, *S. aureus* pathogenicity island, SaPI2R, complete sequence, strain: OC3	AB983196
18.	*S. aureus* DNA, *S. aureus* pathogenicity island, SaPI1, complete sequence, strain: OC3	AB983198
19.	*S. aureus* DNA, *S. aureus* pathogenicity island, SaPI (fhuD), complete sequence, strain: OC3	AB983199
20.	*S. aureus* pathogenicity island SaPIbov, complete sequence	AF217235
21.	*S. aureus* pathogenicity island 3, complete sequence	AF410775

22.	*S. aureus* V329 pathogenicity island SaPIbov2, complete sequence	AY220730
23.	*S. aureus* strain RN3984 mobile pathogenicity island SaPI2, complete sequence	EF010993
24.	*S. aureus* strain BA4 pathogenicity island SaPIbov4, complete sequence	HM211303
25.	*S. aureus* strain JP5338 pathogenicity island SaPIbov5, complete sequence	HM228919
26.	*S. aureus* strain DL584 pathogenicity island SaPIeq1, complete sequence	HM228920
27.	*S. aureus* strain 68111 mobile pathogenicity island SaPI68111, complete sequence	JN689383
28.	*S. aureus* strain 38088 mobile pathogenicity island SaPI2, partial sequence	KP334121
29.	*S. aureus* ED98, pathogenicity island SaPIAv, complete genome	NC_013450

Suppl. tab. 3. An overview of mobile genetic elements (MGEs) occurring in the analysed strains. *Common MGEs*: MGEs known to be typical for poultry strains; *Other plasmids*: other, known up to the date plasmids; *Other prophages*: other prophages, putative, not observed before; *Other PIs*: other pathogenicity islands, besides SaPIeq1, putative, not observed before.

		ch3	ch5	ch9	ch21	ch22	ch23	ch24	pa3	ph2
**Common MGEs**	φAv1	□	□	□	□	□	□	□	□	□
	φAvβ	■	■	■	■	■	■	□	□	□
	pAvX	□	■	■	■	■	■	□	■	■
	pAvY	■	■	■	■	■	■	□	□	□
	SaPIAv	□	□	□	■	■	■	□	□	□
**Other plasmids**	pbc16	□	□	□	□	□	□	□	■	■
	pmccl5	□	□	□	□	□	□	■	□	□
	pph2	□	□	□	□	□	□	□	□	■
	psap074a	■	■	□	□	□	□	□	□	□
	pwbg750	□	□	□	□	□	□	■	□	□
**Other prophages**	φch3	■	□	□	□	□	□	□	□	□
	φch5	□	■	□	□	□	□	□	□	□
	φch9	□	□	■	□	□	□	□	□	□
	φch21	□	□	□	■	■	□	□	□	□
	φch23	□	□	□	□	□	■	□	□	□
	φch24a	□	□	□	□	□	□	■	□	□
	φch24b	□	□	□	□	□	□	■	□	□
	φch24c	□	□	□	□	□	□	■	□	□
	φpa3a	□	□	□	□	□	□	□	■	■
	φpa3b	□	□	□	□	□	□	□	■	■
**Other PIs**	SaPIch9	□	□	■	□	□	□	□	□	□
	SaPIeq1	□	□	□	□	□	□	■	□	□
	SaPIpa3a	□	□	□	□	□	□	□	■	■
	SaPIpa3b	□	□	□	□	□	□	□	■	■

Suppl. tab. 4. Locations of mobile genetic elements (MGEs) as prophages and pathogenicity islands in the analysed strains.

	MGE	Strain	Accession	Beginning	End	Strand
Prophages	φAvβ	ch3	MOYG00000037	94740	141507	plus
		ch5	MSGQ00000041	52888	99130	plus
		ch9	MOYH00000035	43743	90510	plus
		ch21	CP017804	2039557	2086324	plus
		ch22	CP017807	2288960	2335727	plus
		ch23	MOYI00000033	43674	90441	plus
	φch3	ch3	MOYG00000019	16182	56953	plus
	φch5	ch5	MSGQ00000018	19886	55145	plus
	φch9	ch9	MOYH00000007	196546	220721	plus
	φch21	ch21	CP017804	850022	894400	plus
		ch22	CP017807	850428	894770	plus
	φch23	ch23	MOYI00000013	43672	85661	plus
	φch24a	ch24	MOYJ00000008	4562	27853	plus
	φch24b	ch24	MOYJ00000043	18398	45162	minus
	φch24c	ch24	MOYJ00000046	52995	86294	minus
	φpa3a	pa3	MOXP00000008	48523	82185	plus
		ph2	MOYK00000006	26158	59820	plus
	φpa3b	pa3	MOXP00000063	29318	70207	minus
		ph2	MOYK00000059	6650	40928	plus
PIs	SaPIAv	ch21	CP017804	826911	842453	plus
		ch22	CP017807	827317	842859	plus
		ch23	MOYI00000013	20545	36103	plus
	SaPIch9	ch9	MOYH00000016	3955	19155	plus
	SaPIeq1	ch24	MOYJ00000046	110753	125727	minus
	SaPIpa3a	pa3	MOXP00000009	16788	27056	plus
		ph2	MOYK00000007	16788	27056	plus
	SaPIpa3b	pa3	MOXP00000025	3763	17516	plus
		ph2	MOYK00000021	3763	17516	plus

Suppl. tab. 5. Loci within the biggest (170 kbp) duplication in ch22.

No.	Product	Segment 1 loci	Segment 2 loci
1.	Two-component sensor histidine kinase	BJL65_07615	BJL65_08780
2.	DNA-binding response regulator	BJL65_07620	BJL65_08785
3.	Pseudouridine synthase	BJL65_07625	BJL65_08790
4.	SMC-Scp complex subunit ScpB	BJL65_07630	BJL65_08795
5.	Segregation/condensation protein A	BJL65_07635	BJL65_08800
6.	Hypothetical protein	BJL65_07640	BJL65_08805
7.	Site-specific tyrosine recombinase XerD	BJL65_07645	BJL65_08810
8.	Transcriptional repressor	BJL65_07650	BJL65_08815
9.	ADP-ribose pyrophosphatase	BJL65_07655	BJL65_08820
10.	Aldo/keto reductase	BJL65_07660	BJL65_08825
11.	Hypothetical protein	BJL65_07665	BJL65_08830
12.	Short-chain dehydrogenase	BJL65_07670	BJL65_08835
13.	Pyrroline-5-carboxylate reductase	BJL65_07675	BJL65_08840
14.	Ribonuclease Z	BJL65_07680	BJL65_08845
15.	Glucose-6-phosphate dehydrogenase	BJL65_07685	BJL65_08850
16.	AraC family transcriptional regulator	BJL65_07690	BJL65_08855
17.	Glucohydrolase	BJL65_07695	BJL65_08860
18.	LacI family transcriptional regulator	BJL65_07700	BJL65_08865
19.	Glyoxalase	BJL65_07710	BJL65_08870
20.	MFS transporter	BJL65_07715	BJL65_08875
21.	Phosphogluconate dehydrogenase (NADP(+)-dependent, decarboxylating)	BJL65_07720	BJL65_08880
22.	Hypothetical protein	BJL65_07725	BJL65_08885
23.	Hypothetical protein	BJL65_07730	BJL65_08890
24.	Hypothetical protein	BJL65_07735	BJL65_08895
25.	Hypothetical protein	BJL65_07740	BJL65_08900
26.	2-oxoglutarate dehydrogenase	BJL65_07745	BJL65_08905
27.	Alpha-ketoacid dehydrogenase subunit beta	BJL65_07750	BJL65_08910
28.	2-oxoisovalerate dehydrogenase	BJL65_07755	BJL65_08915
29.	Dihydrolipoyl dehydrogenase	BJL65_07760	BJL65_08920
30.	DNA repair protein RecN	BJL65_07765	BJL65_08925
31.	Arginine repressor	BJL65_07770	BJL65_08930
32.	Geranyl transferase	BJL65_07775	BJL65_08935
33.	Exodeoxyribonuclease VII small subunit	BJL65_07780	BJL65_08940
34.	Exodeoxyribonuclease VII large subunit	BJL65_07785	BJL65_08945

No.	Product	Segment 1 loci	Segment 2 loci
35.	N utilization substance protein B	BJL65_07790	BJL65_08950
36.	Hypothetical protein	BJL65_07795	BJL65_08955
37.	Acetyl-CoA carboxylase biotin carboxylase subunit	BJL65_07800	BJL65_08960
38.	Acetyl-CoA carboxylase, biotin carboxyl carrier protein	BJL65_07805	BJL65_08965
39.	Elongation factor P	BJL65_07810	BJL65_08970
40.	Peptidase M24 family protein	BJL65_07815	BJL65_08975
41.	Hypothetical protein	BJL65_07820	BJL65_08980
42.	Hypothetical protein	BJL65_07825	BJL65_08985
43.	Octanoyltransferase	BJL65_07830	BJL65_08990
44.	Rhodanese	BJL65_07835	BJL65_08995
45.	Glycine dehydrogenase (aminomethyl-transferring)	BJL65_07840	BJL65_09000
46.	Glycine dehydrogenase (aminomethyl-transferring)	BJL65_07845	BJL65_09005
47.	Glycine cleavage system protein T	BJL65_07850	BJL65_09010
48.	Shikimate kinase	BJL65_07855	BJL65_09015
49.	Competence protein ComGF	BJL65_07865	BJL65_09020
50.	Competence protein ComGD	BJL65_07875	BJL65_09030
51.	Competence protein ComGE	BJL65_07870	BJL65_09025
52.	Competence protein ComGC	BJL65_07880	BJL65_09035
53.	Competence protein ComGB	BJL65_07885	BJL65_09040
54.	Type II secretion system protein E	BJL65_07890	BJL65_09045
55.	Hydroxyacylglutathione hydrolase	BJL65_07895	BJL65_09050
56.	Hypothetical protein	BJL65_07900	BJL65_09055
57.	Glucokinase	BJL65_07905	BJL65_09060
58.	Hypothetical protein	BJL65_07910	BJL65_09065
59.	Rhomboid family intramembrane serine protease	BJL65_07915	BJL65_09070
60.	5-formyltetrahydrofolate cyclo-ligase	BJL65_07920	BJL65_09075
61.	50S ribosomal protein L33	BJL65_07925	BJL65_09080
62.	Penicillin-binding protein	BJL65_07930	BJL65_09085
63.	Superoxide dismutase	BJL65_07935	BJL65_09090
64.	Transcriptional repressor	BJL65_07940	BJL65_09095
65.	Metal ABC transporter permease	BJL65_07945	BJL65_09100
66.	Zinc ABC transporter ATP-binding protein	BJL65_07950	BJL65_09105
67.	Deoxyribonuclease IV	BJL65_07955	BJL65_09110
68.	DEAD/DEAH box helicase	BJL65_07960	BJL65_09115
69.	Nif3-like dinuclear metal center hexameric protein	BJL65_07965	BJL65_09120
70.	tRNA methyltransferase	BJL65_07970	BJL65_09125

No.	Product	Segment 1 loci	Segment 2 loci
71.	RNA polymerase sigma factor RpoD	BJL65_07975	BJL65_09130
72.	DNA primase	BJL65_07980	BJL65_09135
73.	Phosphoenolpyruvate synthase regulatory protein	BJL65_07985	BJL65_09140
74.	CBS domain-containing protein	BJL65_07990	BJL65_09145
75.	Glycine--tRNA ligase	BJL65_07995	BJL65_09150
76.	DNA repair protein RecO	BJL65_08000	BJL65_09155
77.	GTPase Era	BJL65_08005	BJL65_09160
78.	Cytidine deaminase	BJL65_08010	BJL65_09165
79.	Diacylglycerol kinase	BJL65_08015	BJL65_09170
80.	rRNA maturation RNase YbeY	BJL65_08020	BJL65_09175
81.	Phosphate starvation-inducible protein PhoH	BJL65_08025	BJL65_09180
82.	Iron transporter	BJL65_08030	BJL65_09185
83.	Hypothetical protein	BJL65_08035	BJL65_09190
84.	Serine protease	BJL65_08040	BJL65_09195
85.	30S ribosomal protein S21	BJL65_08045	BJL65_09200
86.	tRNA (N(6)-L-threonylcarbamoyladenosine(37)-C(2))-methylthiotransferase MtaB	BJL65_08050	BJL65_09205
87.	16S rRNA (uracil(1498)-N(3))-methyltransferase	BJL65_08055	BJL65_09210
88.	Ribosomal protein L11 methyltransferase	BJL65_08060	BJL65_09215
89.	Molecular chaperone DnaJ	BJL65_08065	BJL65_09220
90.	Molecular chaperone DnaK	BJL65_08070	BJL65_09225
91.	Nucleotide exchange factor GrpE	BJL65_08075	BJL65_09230
92.	Heat-inducible transcription repressor HrcA	BJL65_08080	BJL65_09235
93.	Coproporphyrinogen III oxidase	BJL65_08085	BJL65_09240
94.	Hypothetical protein	BJL65_08090	BJL65_09245
95.	Elongation factor 4	BJL65_08095	BJL65_09250
96.	30S ribosomal protein S20	BJL65_08100	BJL65_09255
97.	DNA polymerase III subunit delta	BJL65_08105	BJL65_09260
98.	DNA internalization-related competence protein ComEC/Rec2	BJL65_08110	BJL65_09265
99.	ComE operon protein 2	BJL65_08115	BJL65_09270
100.	Competence protein ComE	BJL65_08120	BJL65_09275
101.	Methyltransferase	BJL65_08125	BJL65_09280
102.	Ribosome silencing factor	BJL65_08130	BJL65_09285
103.	HAD family hydrolase	BJL65_08135	BJL65_09290
104.	Nicotinate (nicotinamide) nucleotide adenylyltransferase	BJL65_08140	BJL65_09295
105.	RNA-binding protein	BJL65_08145	BJL65_09300

No.	Product	Segment 1 loci	Segment 2 loci
106.	Shikimate dehydrogenase	BJL65_08150	BJL65_09305
107.	Ribosome biogenesis GTPase YqeH	BJL65_08155	BJL65_09310
108.	Hypothetical protein	BJL65_08160	BJL65_09315
109.	5'-methylthioadenosine/S-adenosylhomocysteine nucleosidase	BJL65_08165	BJL65_09320
110.	Hypothetical protein	BJL65_08175	BJL65_09325
111.	Carboxylate--amine ligase	BJL65_08180	BJL65_09330
112.	Enterotoxin	BJL65_08185	BJL65_09335
113.	Fic/DOC family protein	BJL65_08190	BJL65_09340
114.	Iron transporter	BJL65_08200	BJL65_09350
115.	Lactam utilization protein LamB	BJL65_08205	BJL65_09355
116.	Acetyl-CoA carboxylase biotin carboxylase subunit	BJL65_08210	BJL65_09360
117.	Acetyl-CoA carboxylase biotin carboxyl carrier protein subunit	BJL65_08215	BJL65_09365
118.	Allophanate hydrolase	BJL65_08220	BJL65_09370
119.	Allophanate hydrolase	BJL65_08225	BJL65_09375
120.	Transcription elongation factor GreA	BJL65_08230	BJL65_09380
121.	Uridine kinase	BJL65_08235	BJL65_09385
122.	Protease	BJL65_08240	BJL65_09390
123.	Peptidase U32	BJL65_08245	BJL65_09395
124.	Methyltransferase	BJL65_08250	BJL65_09400
125.	Hypothetical protein	BJL65_08255	BJL65_09405
126.	Holliday junction DNA helicase RuvA	BJL65_08260	BJL65_09410
127.	Hypothetical protein	BJL65_08265	BJL65_09415
128.	Alanine--tRNA ligase	BJL65_08270	BJL65_09420
129.	RecD/TraA family helicase	BJL65_08275	BJL65_09425
130.	Hypothetical protein	BJL65_08280	BJL65_09430
131.	tRNA 2-thiouridine(34) synthase MnmA	BJL65_08285	BJL65_09435
132.	Cysteine desulfurase NifS	BJL65_08290	BJL65_09440
133.	Hypothetical protein	BJL65_08295	BJL65_09445
134.	Hypothetical protein	BJL65_08300	BJL65_09450
135.	CsbD family protein	BJL65_08305	BJL65_09455
136.	Transcriptional regulator	BJL65_08310	BJL65_09460
137.	Recombinase RarA	BJL65_08315	BJL65_09465
138.	tRNA threonylcarbamoyladenosine dehydratase	BJL65_08320	BJL65_09470
139.	Aspartate--tRNA ligase	BJL65_08335	BJL65_09480
140.	Histidine--tRNA ligase	BJL65_08340	BJL65_09485

No.	Product	Segment 1 loci	Segment 2 loci
141.	Cell wall amidase	BJL65_08345	BJL65_09490
142.	D-tyrosyl-tRNA(Tyr) deacylase	BJL65_08350	BJL65_09495
143.	GTP pyrophosphokinase	BJL65_08355	BJL65_09500
144.	Adenine phosphoribosyltransferase	BJL65_08360	BJL65_09505
145.	Single-stranded-DNA-specific exonuclease RecJ	BJL65_08365	BJL65_09510
146.	Protein translocase subunit SecDF	BJL65_08370	BJL65_09515
147.	Preprotein translocase subunit YajC	BJL65_08375	BJL65_09520
148.	tRNA guanosine(34) transglycosylase Tgt	BJL65_08380	BJL65_09525
149.	tRNA preQ1(34) S-adenosylmethionine ribosyltransferase-isomerase QueA	BJL65_08385	BJL65_09530
150.	Holliday junction DNA helicase RuvB	BJL65_08390	BJL65_09535
151.	Holliday junction DNA helicase RuvA	BJL65_08395	BJL65_09540
152.	ACT domain-containing protein	BJL65_08400	BJL65_09545
153.	GTPase ObgE	BJL65_08405	BJL65_09550
154.	50S ribosomal protein L27	BJL65_08410	BJL65_09555
155.	Hypothetical protein	BJL65_08415	BJL65_09560
156.	50S ribosomal protein L21	BJL65_08420	BJL65_09565
157.	Rod shape-determining protein MreD	BJL65_08425	BJL65_09570
158.	Rod shape-determining protein MreC	BJL65_08430	BJL65_09575
159.	Hypothetical protein	BJL65_08435	BJL65_09580
160.	DUF4930 domain-containing protein	BJL65_08440	BJL65_09585
161.	Hypothetical protein	BJL65_08445	BJL65_09590
162.	Hypothetical protein	BJL65_08450	BJL65_09595
163.	Prepilin peptidase	BJL65_08455	BJL65_09600
164.	Bifunctional folylpolyglutamate synthase/dihydrofolate synthase	BJL65_08465	BJL65_09605
165.	Valine--tRNA ligase	BJL65_08470	BJL65_09610
166.	DNA-3-methyladenine glycosylase	BJL65_08475	BJL65_09615
167.	Aminopeptidase	BJL65_08480	BJL65_09620
168.	Glutamate-1-semialdehyde-2,1-aminomutase	BJL65_08490	BJL65_09625
169.	Delta-aminolevulinic acid dehydratase	BJL65_08495	BJL65_09630
170.	Uroporphyrinogen III synthase	BJL65_08500	BJL65_09635
171.	Hydroxymethylbilane synthase	BJL65_08505	BJL65_09640
172.	Cytochrome C assembly protein	BJL65_08510	BJL65_09645
173.	Glutamyl-tRNA reductase	BJL65_08515	BJL65_09650
174.	YihA family ribosome biogenesis GTP-binding protein	BJL65_08520	BJL65_09655
175.	ISL3 family transposase	BJL65_08525	BJL65_09660

Suppl. tab. 6. Loci within the medium (54 kbp) duplication in ch22.

No.	Product	Segment 1 loci	Segment 2 loci
1.	Flap endonuclease	BJL65_07325	BJL65_08530
2.	Dynamin family protein	BJL65_07330	BJL65_08535
3.	Sulfite reductase subunit alpha	BJL65_07335	BJL65_08540
4.	DUF4889 domain-containing protein	BJL65_07345	BJL65_08545
5.	RNA methyltransferase	BJL65_07350	BJL65_08550
6.	Cell division protein GpsB	BJL65_07360	BJL65_08560
7.	Hypothetical protein	BJL65_07365	BJL65_08565
8.	Hypothetical protein	BJL65_07370	BJL65_08570
9.	Holliday junction resolvase RecU	BJL65_07380	BJL65_08575
10.	Transglycosylase	BJL65_07385	BJL65_08580
11.	Hypothetical protein	BJL65_07390	BJL65_08585
12.	Endonuclease III	BJL65_07395	BJL65_08590
13.	DNA replication protein DnaD	BJL65_07400	BJL65_08595
14.	Asparagine--tRNA ligase	BJL65_07405	BJL65_08600
15.	ATP-dependent helicase	BJL65_07410	BJL65_08605
16.	Biotin--[acetyl-CoA-carboxylase	BJL65_07415	BJL65_08610
17.	CCA tRNA nucleotidyltransferase	BJL65_07420	BJL65_08615
18.	N-acetyl-alpha-D-glucosaminyl L-malate synthase BshA	BJL65_07425	BJL65_08620
19.	Nucleotide pyrophosphohydrolase	BJL65_07430	BJL65_08625
20.	Zinc metallopeptidase	BJL65_07435	BJL65_08630
21.	Hypothetical protein	BJL65_07440	BJL65_08635
22.	Hypothetical protein	BJL65_07445	BJL65_08640
23.	Hypothetical protein	BJL65_07450	BJL65_08645
24.	3-phosphoshikimate 1-carboxyvinyltransferase	BJL65_07455	BJL65_08650
25.	3-dehydroquinate synthase	BJL65_07460	BJL65_08655
26.	Chorismate synthase	BJL65_07465	BJL65_08660
27.	Hypothetical protein	BJL65_07470	BJL65_08665
28.	Nucleoside-diphosphate kinase	BJL65_07475	BJL65_08670
29.	Heptaprenyl diphosphate synthase	BJL65_07480	BJL65_08675
30.	Bifunctional demethylmenaquinone methyltransferase/2-methoxy-6-polyprenyl-1,4-benzoquinol methylase	BJL65_07485	BJL65_08680
31.	Heptaprenyl pyrophosphate synthase subunit A	BJL65_07490	BJL65_08685
32.	DNA-binding protein	BJL65_07500	BJL65_08690
33.	Glycerol-3-phosphate dehydrogenase	BJL65_07505	BJL65_08695
34.	RIbosome biogenesis GTPase Der	BJL65_07510	BJL65_08700
35.	30S ribosomal protein S1	BJL65_07515	BJL65_08705

No.	Product	Segment 1 loci	Segment 2 loci
36.	Cytidylate kinase	BJL65_07535	BJL65_08710
37.	L-asparaginase	BJL65_07540	BJL65_08715
38.	Hypothetical protein	BJL65_07545	BJL65_08720
39.	Peptidoglycan-binding protein LysM	BJL65_07555	BJL65_08725
40.	Recombinase RecQ	BJL65_07560	BJL65_08730
41.	Hypothetical protein	BJL65_07565	BJL65_08735
42.	Ferredoxin	BJL65_07570	BJL65_08740
43.	Riboflavin transporter RibU	BJL65_07575	BJL65_08745
44.	Hypothetical protein	BJL65_07580	BJL65_08750
45.	Hypothetical protein	BJL65_07590	BJL65_08755
46.	Hypothetical protein	BJL65_07595	BJL65_08760
47.	Hypothetical protein	BJL65_07600	BJL65_08765
48.	Hypothetical protein	BJL65_07605	BJL65_08770
49.	Hypothetical protein	BJL65_07610	BJL65_08775

Suppl. tab. 7. Loci within the smallest (12 kbp) duplication in ch22.

No.	Product	Segment 1 loci	Segment 2 loci
1.	Transposase	BJL65_09810	BJL65_11310
2.	Hypothetical protein	BJL65_09815	[BJL65_11315]
3.	Hypothetical protein	BJL65_09820	BJL65_11320
4.	Mannosyl-glycoprotein endo-beta-N-acetylglucosamidase	BJL65_09825	BJL65_11325
5.	Hypothetical protein	BJL65_09830	BJL65_11330
6.	Hypothetical protein	BJL65_09835	------------------
7.	Hypothetical protein	------------------	BJL65_11335
8.	Cell division protein FtsK	BJL65_09840	BJL65_11340
9.	Hypothetical protein	BJL65_09845	BJL65_11345
10.	Hypothetical protein	BJL65_09850	BJL65_11350
11.	Hypothetical protein	BJL65_09855	BJL65_11355
12.	AAA family ATPase	BJL65_09860	BJL65_11360
13.	Conjugal transfer protein	BJL65_09865	BJL65_11365
14.	Hypothetical protein	BJL65_09870	BJL65_11370
15.	Conjugal transfer protein	BJL65_09875	BJL65_11375

Suppl. tab. 8. Differentiating loci within the biggest (170 kbp) duplication in ch22.

No.	Product	Segment 1 loci	Segment 2 loci	Protein length		Differences
1.	Competence protein ComGF	BJL65_07865	BJL65_09020	124	165	Shortening of protein the sequence by N-terminal 41 aa.
2.	ISL3 family transposase	BJL65_08525	BJL65_09660	439	439	Protein sequences identical. Completely different sequences 96-200 bp upstream the CDS.

Suppl. tab. 9. Differentiating loci within the smallest (12 kbp) duplication in ch22.

No.	Product	Segment 1 loci	Segment 2 loci	Protein length		Differences
1.	Mannosyl-glycoprotein endo-beta-N-acetylglucosamidase	BJL65_09825	BJL65_11325	342	342	One K67E sense mutation.
2.	Hypothetical protein	BJL65_09830	BJL65_11330	643	643	19 sense mutations.
3.	Cell division protein FtsK	BJL65_09840	BJL65_11340	453	452	8 sense mutation. The latter shortened by the terminal R residue.
4.	Hypothetical protein	BJL65_09850	BJL65_11350	76	76	2 sense mutations.
5.	Hypothetical protein	BJL65_09855	BJL65_11355	72	72	One G61A sense mutation.
6.	AAA family ATPase	BJL65_09860	BJL65_11360	831	831	3 sense mutations.
7.	Conjugal transfer protein	BJL65_09865	BJL65_11365	129	129	5 sense mutations.
8.	Hypothetical protein	BJL65_09870	BJL65_11370	86	86	One I38V sense mutation.
9.	Conjugal transfer protein	BJL65_09875	BJL65_11375	358	358	8 sense mutation.

Suppl. tab. 10. Loci of N315 strain used as queries for phylogenetic analysis of strains belonging to ST5 group.

No.	Locus	Product	Beginning	End	Length
1.	SA0015	dnaC, replicative DNA helicase	21445	21939	495
2.	SA0016	adenylosuccinate synthase	23216	23628	413
3.	SA0022	probable 5[prime]-nucleotidase precursor	31506	31999	494
4.	SA0098	aminoacylase	110529	110948	420
5.	SA0103	conserved hypothetical protein	117750	118187	438
6.	SA0113	ornithine cyclodeaminase	130244	130731	488
7.	SA0120	hypothetical protein	139445	139919	475
8.	SA0123	UDP-glucose 4-epimerase	142173	142618	446
9.	SA0140	phosphatase homologue	161073	161526	454
10.	SA0171	NAD-dependent formate dehydrogenase	194535	195002	468
11.	SA0177	argJ, arginine biosynthesis bifunctional protein	207388	206898	491
12.	SA0182	indole-3-pyruvate decarboxylase	213236	213690	455
13.	SA0202	gamma-glutamyltranspeptidase precursor	239439	239855	417
14.	SA0210	NADH-dependent dehydrogenase	249389	249807	419
15.	SA0220	glycerophosphodiester phosphodiesterase	263008	263471	464
16.	SA0240	sorbitol dehydrogenase	291411	291896	486
17.	SA0256	6-phospho-beta-glucosidase	310060	310488	429
18.	SA0299	carbohydrate kinase, PfkB family	355123	355563	441
19.	SA0311	trimethylamine dehydrogenase	369364	369789	426
20.	SA0321	carbohydrate kinase, PfkB family	377922	378349	428
21.	SA0346	cystathionine beta-lyase	405743	406170	428
22.	SA0361	phosphoglycerate mutase	418661	419144	484
23.	SA0376	GMP synthase	434800	435239	440
24.	SA0419	cystathionine gamma-synthase	480802	481293	492
25.	SA0429	transcription activator of glutamate synthase operon	489886	490342	457
26.	SA0431	NADH-glutamate synthase small subunit	495821	496294	474
27.	SA0449	conserved hypothetical protein	521381	521848	468
28.	SA0501	RNA polymerase beta-prime chain	586357	586786	430
29.	SA0507	N-acyl-L-amino acid amidohydrolase	592338	592835	498
30.	SA0512	branched-chain amino acid aminotroansferase	599411	599841	431
31.	SA0527	glucosamine-6-phosphate isomerase	622181	622659	479
32.	SA0537	phosphomethylpyrimidine kinase	631634	632116	483
33.	SA0572	esterase/lipase	665507	665947	441
34.	SA0646	deoxyribodipyrimidine photolyase	741132	741557	426
35.	SA0671	urea amidolyase	764197	764696	500
36.	SA0689	ferrichrome ABC transporter permease	788137	788636	500
37.	SA0697	glycerate kinase	795048	795547	500
38.	SA0730	phosphoglycerate mutase	836703	837148	446
39.	SA0740	hypothetical protein	847062	847504	443
40.	SA0791	glycerate dehydrogenase	896974	897414	441

41.	SA0817	NADH-dependent flavin oxidoreductase	921075	921477	403
42.	SA0821	argH, argininosuccinate lyase	926505	926012	494
43.	SA0859	thimet oligopeptide	974341	974741	401
44.	SA0896	menD, menaquinone biosynthesis protein	1017529	1017968	440
45.	SA0924	purN, phosphoribosylglycinamide formyltransferase	1049523	1050001	479
46.	SA0945	component of pyruvate dehydrogenase	1073758	1074161	404
47.	SA0956	Mn2+-transpor t protein	1083110	1083534	425
48.	SA0969	glycerophosphoryl diester phosphodiesterase	1099873	1099466	408
49.	SA1044	dihydroorotase	1181330	1181731	402
50.	SA1089	sucD, succinyl-CoA synthetase	1232627	1233078	452
51.	SA1115	ribC, riboflavin kinase / FAD synthase	1264780	1265262	483
52.	SA1142	glpD, aerobic glycerol-3-phosphate dehydrogenase	1299840	1300339	500
53.	SA1150	glnA, glutamine-ammonia ligase	1307593	1308024	432
54.	SA1177	transketolase	1339372	1339785	414
55.	SA1226	aspartate semialdehyde dehydrogenase (ASD)	1401265	1401747	483
56.	SA1308	30S ribosomal protein S1	1513993	1514478	486
57.	SA1334	pyrroline-5-carboxylate reductase	1540736	1541178	443
58.	SA1342	gnd, phosphogluconate dehydrogenase	1551566	1551968	403
59.	SA1366	glycine dehydrogenase subunit 1	1575507	1575017	491
60.	SA1397	cdd, cytidine deaminase	1603292	1603754	463
61.	SA1412	oxygen-independent coproporphyrinogen oxidase III	1617553	1618016	464
62.	SA1460	relA, GTP pyrophosphokinase	1665882	1666352	471
63.	SA1487	folC, folylpolyglutamate synthase	1693720	1693267	454
64.	SA1496	glutamyl-tRNA reductase	1704604	1705096	493
65.	SA1524	malate dehydrogenase homolog	1739845	1739355	491
66.	SA1545	D-3-phosphoglycerate dehydrogenase	1766721	1767216	496
67.	SA1554	acsA, acetyl-CoA synthetase	1781729	1781281	449
68.	SA1585	proline dehydrogenase	1824023	1824497	475
69.	SA1614	menC	1848056	1848544	489
70.	SA1619	hypothetical protein	1853647	1854096	450
71.	SA1669	citG, fumarate hydratase, class-II	1906352	1905900	453
72.	SA1681	glutamate-1-semialdehyde aminotransferase	1926891	1927314	424
73.	SA1735	manganese-dependent inorganic pyrophosphatase	1987761	1988244	484
74.	SA1814	succinyl-diaminopimelate desuccinylase	2053853	2054311	459
75.	SA1843	agrC, accessory gene regulator C	2080947	2081401	455
76.	SA1864	3-isopropylmalate dehydratase large subunit	2105510	2106003	494
77.	SA1923	transcription termination factor Rho	2171616	2172088	473
78.	SA1924	aldehyde dehydrogenase	2173216	2173675	460
79.	SA1965	glmM, femD, phosphoglucosamine-mutase	2226346	2225913	434
80.	SA1991	6-phospho-beta-galactosidase	2263407	2263853	447
81.	SA2008	alsS, alpha-acetolactate synthase	2280729	2280271	459
82.	SA2071	moeB, molybdopterin biosynthesis protein moeB	2332760	2332305	456
83.	SA2099	monooxygenase	2359332	2359800	469
84.	SA2120	amino acid amidohydrolase	2383969	2384377	409

85.	SA2121	hutI, imidazolonepropionase	2386152	2385678	475
86.	SA2214	bioA, aminotransferase	2485384	2485811	428
87.	SA2240	para-nitrobenzyl esterase chain A	2517061	2517511	451
88.	SA2260	glucose 1-dehydrogenase	2538172	2537713	460
89.	SA2304	fructose-bisphosphatase	2589863	2590336	474
90.	SA2327	pyruvate oxidase	2613400	2612925	476
91.	SA2348	crtN, squalene desaturase	2638864	2638403	462
92.	SA2393	2-dehydropantoate 2-reductase	2680864	2681340	477
93.	SA2410	anaerobic ribonucleoside-triphosphate reductase	2701426	2701886	461
94.	SA2439	sasF, hypothetical protein	2743554	2743068	487
95.	SA2445	hypothetical protein	2753616	2753158	459
96.	SA2470	histidinol dehydrogenase	2781905	2782326	422
97.	SA2490	pckA, phosphoenolpyruvate carboxykinase	1844849	1845280	432

Suppl. tab. 11. List of genomic assemblies of *S. aureus* strains used as ST5 representatives. Reference strains N315 and ED98 are placed at the beginning of the table; ST5 strains analysed in this study, at the end.

No.	Assembly version	Strain	Biosample	Country	Year	Host
1.	GCA_000009645.1	N315	SAMD00061099	Japan	1982	Human
2.	GCA_000024585.1	ED98	SAMN02604165	Northern Ireland	1996	Poultry
3.	GCA_000253135.1	ECT-R_2	SAMEA2271964	Sweden	2005	Human
4.	GCA_000360905.1	M0351	SAMN00809176	USA	2003	Human
5.	GCA_000361585.1	M0580	SAMN00809058	USA	2004	Human
6.	GCA_000362525.1	M1036	SAMN00809053	USA	2004	Human
7.	GCA_000363385.1	M1359	SAMN00809082	USA	2004	Human
8.	GCA_000390205.1	06BA18369	SAMN02470878	Canada	2006	Human
9.	GCA_000401435.1	091751	SAMN02470727	Switzerland	2001	Human
10.	GCA_000442355.1	SA16	SAMN02179359	Brazil	2009	Human
11.	GCA_000531065.1	M0167	SAMN02325670	USA	2003	Human
12.	GCA_000532195.1	M0962	SAMN02325847	USA	2004	Human
13.	GCA_000536415.1	M1558	SAMN02364069	USA	2004	Human
14.	GCA_000543825.1	M0798	SAMN02325710	USA	2004	Human
15.	GCA_000544385.1	M0841	SAMN02325740	USA	2004	Human
16.	GCA_000544745.1	M0875	SAMN02325775	USA	2004	Human
17.	GCA_000545985.1	M0972	SAMN02325856	USA	2004	Human
18.	GCA_000546085.1	M0983	SAMN02334620	USA	2004	Human
19.	GCA_000546225.1	M0997	SAMN02334630	USA	2004	Human
20.	GCA_000547145.1	M1087	SAMN02334690	USA	2004	Human
21.	GCA_000547585.1	M1580	SAMN02339955	USA	2005	Human
22.	GCA_000548225.1	M1395	SAMN02350571	USA	2004	Human
23.	GCA_000548265.1	M1397	SAMN02350573	USA	2004	Human
24.	GCA_000550365.1	M1523	SAMN02364041	USA	2004	Human
25.	GCA_000551885.1	M0084	SAMN02325170	USA	2003	Human
26.	GCA_000558085.1	M0609	SAMN02325527	USA	2004	Human
27.	GCA_000558265.1	M0619	SAMN02325536	USA	2004	Human
28.	GCA_000559505.1	H83536	SAMN02383625	USA	2011	Human
29.	GCA_000561705.1	T78544	SAMN02383750	USA	2011	Human
30.	GCA_000570235.1	F53388	SAMN02385472	USA	2012	Human
31.	GCA_000571115.1	M46070	SAMN02385409	USA	2012	Human
32.	GCA_000571635.1	F77919	SAMN02385378	USA	2012	Human
33.	GCA_000574725.1	M1220	SAMN02360878	USA	2004	Human
34.	GCA_000576815.1	M12709	SAMN02383703	USA	2011	Human
35.	GCA_000578455.1	H38226	SAMN02398771	USA	2012	Human
36.	GCA_000579115.1	W73676	SAMN02398815	USA	2012	Human
37.	GCA_000586675.1	DAR5814	SAMN02403910	Colombia	2002	Human
38.	GCA_000594945.1	DAR126	SAMN02403938	Finland	1992	Human

39.	GCA_000594965.1	DAR116	SAMN02403937	UK	1980	Human
40.	GCA_000594985.1	DAR104	SAMN02403936	Denmark	1964	Human
41.	GCA_000595025.1	DAR5789	SAMN02403934	Colombia	2001	Human
42.	GCA_000595045.1	DAR5790	SAMN02403933	Colombia	2001	Human
43.	GCA_000595065.1	DAR5791	SAMN02403932	Colombia	2001	Human
44.	GCA_000595085.1	DAR5792	SAMN02403931	Colombia	2001	Human
45.	GCA_000595105.1	DAR5793	SAMN02403930	Colombia	2001	Human
46.	GCA_000595125.1	DAR5794	SAMN02403929	Colombia	2001	Human
47.	GCA_000595145.1	DAR5795	SAMN02403928	Colombia	2001	Human
48.	GCA_000595165.1	DAR5796	SAMN02403927	Colombia	2001	Human
49.	GCA_000595185.1	DAR5797	SAMN02403926	Colombia	2001	Human
50.	GCA_000595205.1	DAR5798	SAMN02403925	Colombia	2001	Human
51.	GCA_000595225.1	DAR5799	SAMN02403924	Colombia	2001	Human
52.	GCA_000595265.1	DAR5801	SAMN02403922	Colombia	2001	Human
53.	GCA_000595285.1	DAR5802	SAMN02403921	Colombia	2001	Human
54.	GCA_000595305.1	DAR5803	SAMN02403920	Colombia	2001	Human
55.	GCA_000595325.1	DAR5804	SAMN02403919	Colombia	2001	Human
56.	GCA_000595345.1	DAR5805	SAMN02403918	Colombia	2001	Human
57.	GCA_000595365.1	DAR5806	SAMN02403917	Colombia	2001	Human
58.	GCA_000595385.1	DAR5807	SAMN02403916	Colombia	2001	Human
59.	GCA_000595405.1	DAR5808	SAMN02403915	Colombia	2001	Human
60.	GCA_000595425.1	DAR5809	SAMN02403914	Colombia	2001	Human
61.	GCA_000595445.1	DAR5811	SAMN02403913	Colombia	2001	Human
62.	GCA_000595465.1	DAR5812	SAMN02403912	Colombia	2001	Human
63.	GCA_000595485.1	DAR5813	SAMN02403911	Colombia	2002	Human
64.	GCA_000595505.1	DAR5815	SAMN02403909	Colombia	2002	Human
65.	GCA_000595525.1	DAR5816	SAMN02403908	Colombia	2002	Human
66.	GCA_000595545.1	DAR5817	SAMN02403907	Colombia	2002	Human
67.	GCA_000595565.1	DAR5819	SAMN02403905	Colombia	2006	Human
68.	GCA_000595585.1	DAR5820	SAMN02403904	Colombia	2006	Human
69.	GCA_000595605.1	DAR5821	SAMN02403903	Colombia	2006	Human
70.	GCA_000595625.1	DAR5822	SAMN02403902	Colombia	2006	Human
71.	GCA_000595645.1	DAR5823	SAMN02403901	Colombia	2006	Human
72.	GCA_000595665.1	DAR5824	SAMN02403900	Colombia	2006	Human
73.	GCA_000595685.1	DAR5825	SAMN02403899	Colombia	2006	Human
74.	GCA_000595745.1	DAR5828	SAMN02403896	Colombia	2006	Human
75.	GCA_000595765.1	DAR5829	SAMN02403895	Colombia	2006	Human
76.	GCA_000595785.1	DAR5830	SAMN02403894	Colombia	2007	Human
77.	GCA_000595805.1	DAR5831	SAMN02403893	Colombia	2006	Human
78.	GCA_000595825.1	DAR5832	SAMN02403892	Colombia	2006	Human
79.	GCA_000595865.1	DAR5834	SAMN02403890	Colombia	2006	Human
80.	GCA_000595885.1	DAR5835	SAMN02403889	Colombia	2006	Human
81.	GCA_000595905.1	DAR5836	SAMN02403888	Colombia	2006	Human
82.	GCA_000595945.1	DAR5838	SAMN02403886	Colombia	2006	Human
83.	GCA_000595965.1	DAR5839	SAMN02403885	Colombia	2007	Human

84.	GCA_000595985.1	DAR5840	SAMN02403884	Colombia	2006	Human
85.	GCA_000596005.1	DAR5841	SAMN02403883	Colombia	2007	Human
86.	GCA_000596085.1	DAR5847	SAMN02403879	Peru	2006	Human
87.	GCA_000596105.1	DAR5848	SAMN02403878	Peru	2006	Human
88.	GCA_000596125.1	DAR5849	SAMN02403877	Peru	2007	Human
89.	GCA_000596145.1	DAR5850	SAMN02403876	Peru	2007	Human
90.	GCA_000596185.1	DAR5853	SAMN02403873	Ecuador	2007	Human
91.	GCA_000596205.1	DAR5854	SAMN02403872	Ecuador	2006	Human
92.	GCA_000596225.1	DAR5856	SAMN02403870	Venezuela	2006	Human
93.	GCA_000596245.1	DAR5857	SAMN02403869	Venezuela	2006	Human
94.	GCA_000596265.1	DAR5858	SAMN02403868	Venezuela	2007	Human
95.	GCA_000596285.1	DAR5859	SAMN02403867	Venezuela	2008	Human
96.	GCA_000596305.1	DAR5861	SAMN02403866	Venezuela	2007	Human
97.	GCA_000596325.1	DAR5862	SAMN02403865	Venezuela	2008	Human
98.	GCA_000596345.1	DAR5863	SAMN02403864	Venezuela	2007	Human
99.	GCA_000596365.1	DAR5864	SAMN02403863	Venezuela	2007	Human
100.	GCA_000596385.1	DAR5865	SAMN02403862	Argentina	2013	Human
101.	GCA_000596485.1	DAR3507	SAMN02403666	USA	2009	Human
102.	GCA_000596565.1	F63704	SAMN02398732	USA	2012	Human
103.	GCA_000605105.1	H43700	SAMN02402522	USA	2013	Human
104.	GCA_000605385.1	M54162	SAMN02402540	USA	2013	Human
105.	GCA_000607585.1	DAR3578	SAMN02403684	USA	2007	Human
106.	GCA_000607645.1	DAR3584	SAMN02403687	USA	2007	Human
107.	GCA_000607685.1	DAR3587	SAMN02403689	USA	2004	Human
108.	GCA_000608925.1	DAR5880	SAMN02403753	Peru	2011	Human
109.	GCA_000608945.1	DAR22	SAMN02403754	Belgium	1995	Human
110.	GCA_000608965.1	DAR5878	SAMN02403755	Peru	2011	Human
111.	GCA_000609005.1	DAR5875	SAMN02403757	Brazil	2012	Human
112.	GCA_000609025.1	DAR5874	SAMN02403758	Colombia	2011	Human
113.	GCA_000609045.1	DAR5873	SAMN02403759	Colombia	2011	Human
114.	GCA_000609945.1	DAR1890	SAMN02403804	USA	2007	Human
115.	GCA_000610405.1	DAR3143	SAMN02403828	Argentina	2005	Human
116.	GCA_000610425.1	DAR3144	SAMN02403829	Argentina	2005	Human
117.	GCA_000610445.1	DAR3148	SAMN02403830	Argentina	2005	Human
118.	GCA_000610465.1	DAR3150	SAMN02403831	Argentina	2005	Human
119.	GCA_000610485.1	DAR3152	SAMN02403832	Argentina	2005	Human
120.	GCA_000610505.1	DAR3153	SAMN02403833	Argentina	2005	Human
121.	GCA_000610565.1	DAR3162	SAMN02403836	Argentina	2006	Human
122.	GCA_000610585.1	DAR3163	SAMN02403837	Argentina	2004	Human
123.	GCA_000610625.1	DAR3175	SAMN02403840	Argentina	2005	Human
124.	GCA_000610645.1	DAR3176	SAMN02403841	Argentina	2006	Human
125.	GCA_000610665.1	DAR3177	SAMN02403842	Argentina	2006	Human
126.	GCA_000610685.1	DAR3178	SAMN02403843	Argentina	2006	Human
127.	GCA_000610705.1	DAR3179	SAMN02403844	Argentina	2005	Human
128.	GCA_000610725.1	DAR3183	SAMN02403845	Argentina	2004	Human

129.	GCA_000610745.1	DAR3198	SAMN02403846	Argentina	2000	Human
130.	GCA_000610765.1	DAR3236	SAMN02403847	Argentina	2006	Human
131.	GCA_000610785.1	DAR5890	SAMN02403848	Venezuela	2012	Human
132.	GCA_000610805.1	DAR5889	SAMN02403849	Peru	2011	Human
133.	GCA_000610825.1	DAR5888	SAMN02403850	Peru	2011	Human
134.	GCA_000610845.1	DAR5887	SAMN02403851	Peru	2011	Human
135.	GCA_000610865.1	DAR5886	SAMN02403852	Peru	2011	Human
136.	GCA_000610885.1	DAR5885	SAMN02403853	Peru	2011	Human
137.	GCA_000610905.1	DAR5884	SAMN02403854	Peru	2011	Human
138.	GCA_000610925.1	DAR5881	SAMN02403855	Peru	2011	Human
139.	GCA_000610945.1	DAR5872	SAMN02403856	Chile	2012	Human
140.	GCA_000610965.1	DAR5871	SAMN02403857	Chile	2012	Human
141.	GCA_000610985.1	DAR5870	SAMN02403858	Chile	2012	Human
142.	GCA_000611005.1	DAR5869	SAMN02403859	Chile	2012	Human
143.	GCA_000611025.1	DAR5868	SAMN02403860	Argentina	2013	Human
144.	GCA_000932245.1	P180	SAMN03253089	Malaysia	2012	Human
145.	GCA_000932275.1	P151	SAMN03253057	Malaysia	2012	Human
146.	GCA_001019065.2	FDAARGOS_37	SAMN03255476	Japan	1996	Human
147.	GCA_001019305.2	NRS70	SAMN03255448	Japan	1982	Human
148.	GCA_001019435.2	NRS1	SAMN03255464	Japan	1996	Human
149.	GCA_001019575.2	HPV107	SAMN03255486	Portugal	1992	Human
150.	GCA_001184345.2	V1127	SAMN03301669	Korea	2014	Human
151.	GCA_001281145.1	SA564	SAMN03267711	Switzerland	2013	Human
152.	GCA_001297285.1	BU_W7A_t11	SAMN03658594	Ghana	2013	Human
153.	GCA_001297525.1	BU_W22_t4	SAMN03658593	Ghana	2013	Human
154.	GCA_001306505.1	HOU1445-VS	SAMN03092872	Brazil	2012	Human
155.	GCA_001306525.1	HOU1444-VR	SAMN03093451	Brazil	2012	Human
156.	GCA_001411615.1	CC072	SAMN03988826	Brazil	2011	Human
157.	GCA_001411635.1	CC017	SAMN03968919	Brazil	2011	Human
158.	GCA_001618305.1	RIVM6519	SAMN04590098	Netherlands	2012	Human
159.	GCA_001640885.1	ZJ5499	SAMN03742530	China	2010	Human
160.	GCA_001640905.1	NCCP14558	SAMN04219574	Korea	2013	Human
161.	GCA_001640925.1	NCCP14562	SAMN03955396	Korea	2013	Human
162.	GCA_001681005.1	Sa14-003	SAMN05188400	Australia	2014	Bovine
163.	GCA_001725965.1	FORC_027	SAMN04046233	Korea	2006	Human
164.	GCA_001899895.1	SA_103	SAMN05858801	Spain	2011	Human
165.	GCA_001900065.1	SA_80004	SAMN05860625	Spain	2014	Human
166.	GCA_001921695.1	C80	SAMN06077115	Brazil	2012	Human
167.	GCA_001975045.1	UCI_28	SAMN06046106	USA	2009	Human
168.	GCA_002094085.1	3503-1	SAMN05383889	China	2014	Human
169.	GCA_002094105.1	4126-1	SAMN05383987	China	2013	Human
170.	GCA_002094135.1	4126-2	SAMN05383989	China	2013	Human
171.	GCA_002121225.1	CFSA314	SAMN06698242	USA	2015	Human
172.	GCA_002121365.1	CFSA304	SAMN06698233	USA	2015	Human
173.	GCA_002123175.1	CFSA210	SAMN06698152	USA	2015	Human

174.	GCA_002125105.1	CFSA107	SAMN06698078	USA	2014	Human
175.	GCA_002125185.1	CFSA084	SAMN06698057	USA	2014	Human
176.	GCA_002125515.1	CFSA041	SAMN06698028	USA	2014	Human
177.	GCA_002131825.1	HU09-1102016	SAMN06163085	Japan	2009	Human
178.	GCA_002204555.1	ISU935	SAMN04571767	USA	2010	Porcine
179.	GCA_002260015.1	UB345	SAMN05721737	Brazil	2012	Human
180.	GCA_002260165.1	UCL409	SAMN05721744	Chile	2012	Human
181.	GCA_002260175.1	UCL720	SAMN05721745	Chile	2014	Human
182.	GCA_002260225.1	UG982	SAMN05721750	Guatemala	2012	Human
183.	GCA_002260275.1	UP109	SAMN05721755	Peru	2011	Human
184.	GCA_002260315.1	UP954	SAMN05721757	Peru	2012	Human
185.	GCA_002260355.1	UG1092	SAMN05721747	Guatemala	2013	Human
186.	GCA_002260385.1	UG258	SAMN05721748	Guatemala	2012	Human
187.	GCA_002260425.1	UP1072	SAMN05721753	Peru	2013	Human
188.	GCA_002267075.1	UC16	SAMN03940720	Colombia	2011	Human
189.	GCA_002267385.1	UV165	SAMN03940728	Venezuela	2012	Human
190.	GCA_002267495.1	UG268	SAMN03940737	Guatemala	2012	Human
191.	GCA_002267605.2	UCL437	SAMN03940748	Chile	2012	Human
192.	GCA_002267645.1	UB590	SAMN03940752	Brazil	2012	Human
193.	GCA_002267705.1	UB594	SAMN03940753	Brazil	2012	Human
194.	GCA_002267765.1	UMX777	SAMN03940759	Mexico	2012	Human
195.	GCA_002267855.1	UG944	SAMN03940770	Guatemala	2012	Human
196.	GCA_002267865.1	UG993	SAMN03940771	Guatemala	2012	Human
197.	GCA_002267935.1	UV1155	SAMN03940775	Venezuela	2013	Human
198.	GCA_002267965.1	UB670	SAMN03944932	Brazil	2012	Human
199.	GCA_002268035.1	UP788	SAMN03940760	Peru	2013	Human
200.	GCA_002268065.1	UG834	SAMN03940762	Guatemala	2013	Human
201.	GCA_002268085.1	UA913	SAMN03940763	Argentina	2013	Human
202.	GCA_002268125.1	UA925	SAMN03940766	Argentina	2013	Human
203.	GCA_002268185.1	UP1027	SAMN03940773	Peru	2013	Human
204.	GCA_002268195.1	UB599	SAMN03944930	Brazil	2012	Human
205.	GCA_002734305.1	A2	SAMN03766003	Brazil	2014	Human
206.	GCA_002734325.1	A10	SAMN03766017	Brazil	2014	Human
207.	GCA_002734345.1	B2	SAMN03766018	Brazil	2014	Human
208.	GCA_002734385.1	B10	SAMN03766021	Brazil	2014	Human
209.	GCA_002734415.1	C6	SAMN03766024	Brazil	2014	Human
210.	GCA_002734425.1	C7	SAMN03766025	Brazil	2014	Human
211.	GCA_002734485.1	B7	SAMN03766020	Brazil	2014	Human
212.	GCA_002734495.1	B6	SAMN03766019	Brazil	2014	Human
213.	GCA_002734505.1	C2	SAMN03766022	Brazil	2014	Human
214.	GCA_002736805.1	SA43	SAMN03115001	Brazil	2009	Human
215.	GCA_002741955.1	VA102_B0	SAMN05853039	USA	2012	Human
216.	GCA_002905355.1	N34_260215	SAMN04089992	Ghana	2015	Human
217.	GCA_002905635.1	W31_250215	SAMN04089993	Ghana	2015	Human
218.	GCA_900017775.1	NZAK3	SAMEA3733527	New Zealand	2005	Human

219.	GCA_900018225.1	st1307	SAMEA1469581	UK	2006	Human
220.	GCA_900018695.1	st326	SAMEA1464437	UK	2002	Human
221.	GCA_900019145.1	st659	SAMEA1464480	UK	2003	Human
222.	GCA_900020455.1	st1664	SAMEA1464719	UK	2007	Human
223.	GCA_900038245.1	st228	SAMEA1464466	UK	2001	Human
224.	GCA_900038765.1	st527	SAMEA1317575	UK	2003	Human
225.	GCA_900039685.1	st586	SAMEA1464299	UK	2003	Human
226.	GCA_900040815.1	st45	SAMEA1317462	UK	2001	Human
227.	GCA_900041065.1	st1827	SAMEA1317405	Ireland	2008	Human
228.	GCA_900041215.1	st2293	SAMEA1469613	UK	2009	Human
229.	GCA_900041995.1	st970	SAMEA1317493	UK	2004	Human
230.	GCA_900042495.1	st774	SAMEA1464454	UK	2004	Human
231.	GCA_900044345.1	st124	SAMEA1464181	UK	2001	Human
232.	GCA_900044435.1	st48	SAMEA1317583	UK	2001	Human
233.	GCA_900045705.1	st277	SAMEA1464600	UK	2002	Human
234.	GCA_900045985.1	st776	SAMEA1464217	UK	2004	Human
235.	GCA_900046145.1	st1792	SAMEA1317469	UK	2008	Human
236.	GCA_900051355.1	st1062	SAMEA1464639	UK	2005	Human
237.	GCA_900052645.1	st2801	SAMEA1464381	UK	2010	Human
238.	GCA_900056305.1	st1815	SAMEA1464546	Ireland	2008	Human
239.	GCA_900058105.1	st2661	SAMEA1464475	UK	2009	Human
240.	GCA_900081725.1	MSSA	SAMEA2384407	USA	2009	Human
241.	GCA_900082665.1	MRSA	SAMEA2384796	USA	2004	Human
242.	GCA_900082675.1	MRSA	SAMEA2384835	USA	2009	Human
243.	GCA_900082885.1	MRSA	SAMEA2384865	USA	2004	Human
244.	GCA_900097405.1	078H	SAMEA3109330	Tanzania	2014	Human
245.	GCA_900097555.1	065H	SAMEA3109329	Tanzania	2014	Human
246.	GCA_900097675.1	MRSA041776/938686	SAMEA2298743	Tanzania	2013	Human
247.	GCA_900097715.1	MRSA012T(+)	SAMEA2298744	Tanzania	2012	Human
248.	GCA_900097735.1	MRSA036667	SAMEA2298739	Tanzania	2012	Human
249.	GCA_900097775.1	MRSA08	SAMEA2298736	Tanzania	2012	Human
250.	GCA_900097815.1	MRSA933832	SAMEA2298707	Tanzania	2012	Human
251.	GCA_900124835.1	3688STDY6124990	SAMEA3449000	Thailand	2015	Human
252.	GCA_900124855.1	3688STDY6124995	SAMEA3449005	Thailand	2015	Human
253.	GCA_900124915.1	3688STDY6125000	SAMEA3449010	Thailand	2015	Human
254.	GCA_900126025.1	3688STDY6124906	SAMEA3448917	Thailand	2015	Human
255.	GCA_900126165.1	3688STDY6124923	SAMEA3448933	Thailand	2015	Human
256.	GCA_900127725.1	3688STDY6125002	SAMEA3449012	Thailand	2015	Human
257.	GCA_900155765.1	CHUV_14	SAMEA48341668	Switzerland	2014	Human
258.	GCA_900250195.1	H1327	SAMEA104473368	Denmark	2014	Human
259.	GCA_900250215.1	H1349	SAMEA104473369	Denmark	2014	Human
260.	GCA_900250225.1	H1326	SAMEA104473367	Denmark	2014	Human
261.	GCA_900250355.1	H1401	SAMEA104473373	Denmark	2014	Human
262.	GCA_900250925.1	M2890	SAMEA104473437	Denmark	2014	Human
263.	GCA_900250935.1	M2943	SAMEA104473442	Denmark	2014	Human

264.	GCA_900251165.1	M3108	SAMEA104473458	Denmark	2014	Human
265.	GCA_900251245.1	M3122	SAMEA104473462	Denmark	2014	Human
266.	GCA_900251295.1	M3330	SAMEA104473476	Denmark	2014	Human
267.	GCA_900251335.1	M3267	SAMEA104473473	Denmark	2014	Human
268.	GCA_900251465.1	M3478	SAMEA104473486	Denmark	2014	Human
269.	GCA_900251505.1	M3401	SAMEA104473480	Denmark	2014	Human
270.	GCA_900251555.1	M3549	SAMEA104473495	Denmark	2014	Human
271.	GCA_900251585.1	M3687	SAMEA104473508	Denmark	2014	Human
272.	GCA_900251655.1	M3626	SAMEA104473505	Denmark	2014	Human
273.	GCA_900251725.1	M3833	SAMEA104473521	Denmark	2014	Human
274.	GCA_900251755.1	M3775	SAMEA104473517	Denmark	2014	Human
275.	GCA_900251865.1	M3868	SAMEA104473525	Denmark	2014	Human
276.	GCA_900251895.1	M3925	SAMEA104473535	Denmark	2014	Human
277.	GCA_900251915.1	M3931	SAMEA104473537	Denmark	2014	Human
278.	GCA_900252025.1	M3991	SAMEA104473543	Denmark	2014	Human
279.	GCA_900252195.1	RH228	SAMEA104473559	Denmark	2014	Human
280.	GCA_003336625.1	CH3	SAMN05853510	Belgium	1976	Poultry
281.	GCA_003336635.1	CH5	SAMN05853511	Belgium	1976	Poultry
282.	GCA_003336495.1	CH9	SAMN05853512	USA	1999	Poultry
283.	GCA_003343155.1	CH21	SAMN05853507	Poland	2008	Poultry
284.	GCA_003350605.1	ch22	SAMN05853508	Poland	2008	Poultry
285.	GCA_003336545.1	CH23	SAMN05853513	Poland	2008	Poultry

Suppl. tab. 12. Number of SNPs between strains analyzed in the study.

	CH21	ch22	CH23	ch24	CH3	CH5	CH9	N315	pa3	ph2	ED98
**CH21**	0	85	278	19522	355	349	345	823	21100	21127	143
**ch22**	85	0	233	19492	311	305	300	779	21070	21097	98
**CH23**	278	233	0	19477	300	298	289	768	21057	21084	165
**ch24**	19522	19492	19477	0	19385	19384	19451	19394	20875	20861	19418
**CH3**	355	311	300	19385	0	108	273	663	20963	20988	243
**CH5**	349	305	298	19384	108	0	273	665	20965	20990	237
**CH9**	345	300	289	19451	273	273	0	737	21021	21048	227
**N315**	823	779	768	19394	663	665	737	0	20959	20984	709
**pa3**	21100	21070	21057	20875	20963	20965	21021	20959	0	165	20997
**ph2**	21127	21097	21084	20861	20988	20990	21048	20984	165	0	21022
**ED98**	143	98	165	19418	243	237	227	709	20997	21022	0

Suppl. tab. 13. List of proteins differentially expressed by VIR CH21 and NVIR ch22 strains as identified by proteomics.

		Locus tags in CH21 and ch22	Protein	Location	Strain	Acronym	MW [kDa]	pI	Score	Peptides	SC [%]
1	1	BJL64_13565<br>BJL65_14845	Diapolycopene oxygenase	in	CH21	CrtP	57.1	7.8	293.8	10	24.3
2	2	BJL64_11045<br>BJL65_12315	Bifunctional hydroxymethylpyrimidine kinase/ phosphomethylpyrimidine kinase	in	CH21	ThiD/J	30.3	5.9	183.6	5	26.8
3	3	BJL64_01840<br>BJL65_01835	NAD(P)-dependent oxidoreductase	in	CH21	3β-HSD	31.8	5.9	324.3	7	33.5
4	4	BJL64_14305<br>BJL65_15585	Arylamine N-acetyltransferase	in	CH21	NhoA	30.7	6	197.1	4	19.2
5	5	BJL64_11040<br>BJL65_12310	Hydroxyethylthiazole kinase	in	CH21	ThiM	28.0	4.4	181.5	5	20.5
6	6	BJL64_09170<br>BJL65_10335	Translaldolase	in	CH21	TalA	25.7	4.6	281.9	8	40.5
7	7	BJL64_09115<br>BJL65_10280	Riboflavin synthase subunit alpha	in	CH21	RibE	23.3	6.3	171.4	5	252
8	8	BJL64_00690<br>BJL65_00690	Hypothetical protein	in	CH21	Hyp	19.2	4.5	356.8	7	34.1
9	9	BJL64_01760<br>BJL65_01755	Peroxiredoxin	in	CH21	AhpC	21.2	4.7	273.8	5	40.2
10	10	BJL64_11525<br>BJL65_12795	Alkaline shock protein	in	CH21	Asp23	19.2	5.0	674.9	12	66.3
11	11	BJL64_00965<br>BJL65_00965	Formate C-acetyltransferase	in	ch22	PflD	84.8	5.2	165.6	4	7.3
12	12	BJL64_08575<br>BJL65_09740	Type I glyceraldehyde-3-phosphate dehydrogenase	in	ch22	G3p2	37	5.9	1002.2	17	49.6
13	13	BJL64_12445<br>BJL65_13715	Hemin ABC transporter ATP binding protein	in	ch22	HrtA1	24.6	5.6	111.0	4	23.5
14	1	BJL64_09350<br>BJL65_10515	DUF4888 domain-containing protein	out	CH21	DUF488	23.7	9.6	1631	21	61.3
15	2	BJL64_02695<br>BJL65_02685	MSCRAMM family adhesion SdrE	out	ch22	SdrE	124	4.1	396	7	11.9
16	3	BJL64_07905<br>BJL65_09090	Superoxide dismutase	out	ch22	SodM1	22.7	5	215.8	4	32.2
17	4	BJL64_01760<br>BJL65_01755	Peroxiredoxin	out	ch22	AhpC	21	4.7	508.4	7	40.7

18	5	BJL64_03485 BJL65_03480	Glycerol phosphate lipoteichoicacid synthase	out	ch22	LtaS	74.4	9.4	1436.6	22	32
19	6	BJL64_01445 BJL65_01440	Lipase2	out	ch22	Lip2	76.5	9.4	1167.1	16	34.4
20	7	BJL64_11645 BJL65_1291	Toxin	out	ch22		15.4	9.7	508.3	7	38.3
21	8	BJL64_14165 BJL65_15445	Lipase1	out	ch22	Lip1	76.5	6.6	1539.2	26	34.4
21	1	BJL64_02580 BJL65_02570	50S ribosomal protein L7/L12	surf	CH21	RL7/12	12.7	4.5	53.9	2	22.1
22	2	BJL64_12760 BJL65_14035	Immunoglobulin-binding protein Sbi	surf	CH21	Sbi	50	9.7	196	4	8.7
23	3	BJL64_14035 BJL65_15315	N-acetylmuramoyl-L-alanine amidase	surf	CH21	Y2979	69.2	5.9	199.7	7	17.3
24	4	BJL64_03805 BJL65_03800	Phosphopyruvate hydratase	surf	ch22	Eno	47.1	4.4	84.5	2	7.6
25	5	BJL64_07465 BJL65_08690	DNA-binding protein	surf	ch22	HU	9.6	10.0	110.7	2	34.4
26	6	BJL64_11780 BJL65_13050	30S ribosomal protein S5	surf	ch22	RS5	17.7	10.3	86.4	2	15.7
27	7	BJL64_13785 BJL65_15065	Fructose bisphosphate aldolase	surf	ch22	Alf2	32.9	4.9	74.2	2	10.1
28	8	BJL64_14045 BJL65_15325	Adhesin (surface protein F)	surf	ch22	SasF	72.1	9.4	93.2	2	6.8

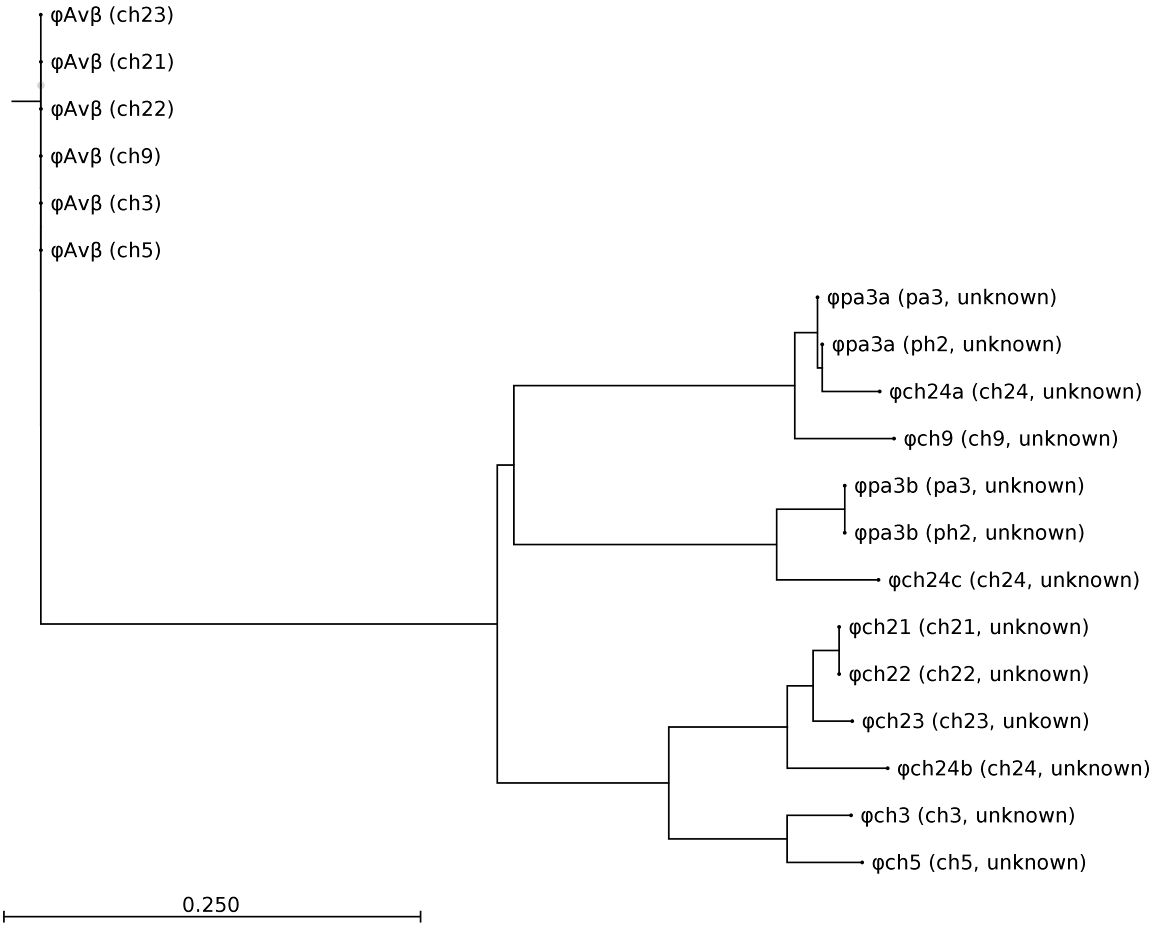

Suppl. fig. 1. Phylogenetic relations among observed prophages.

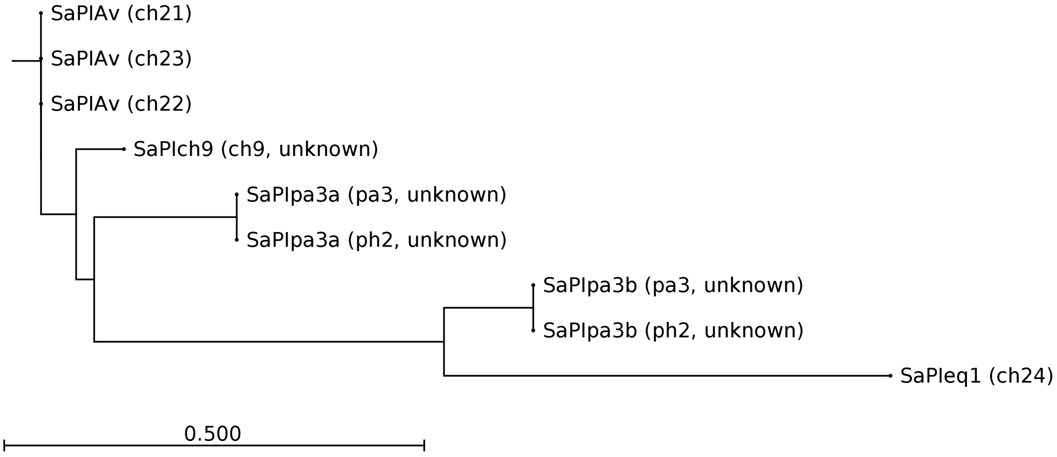

Suppl. fig. 2. Phylogenetic relations among observed pathogenicity islands.

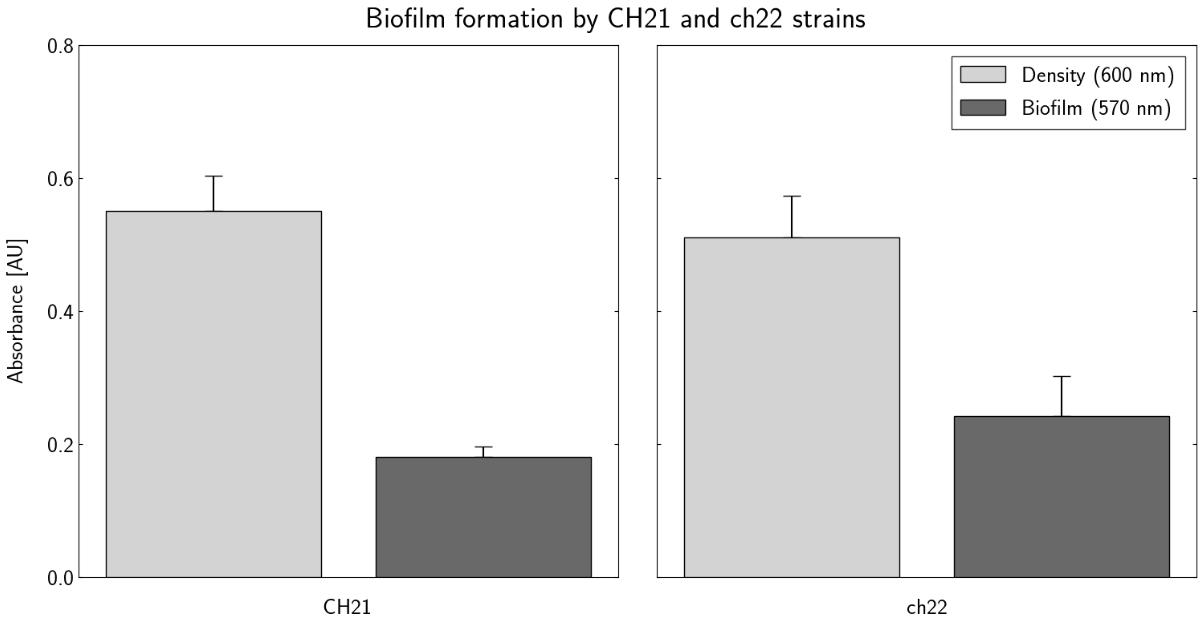

Suppl. fig. 3. Biofilm formation by CH21 and ch22 strains.

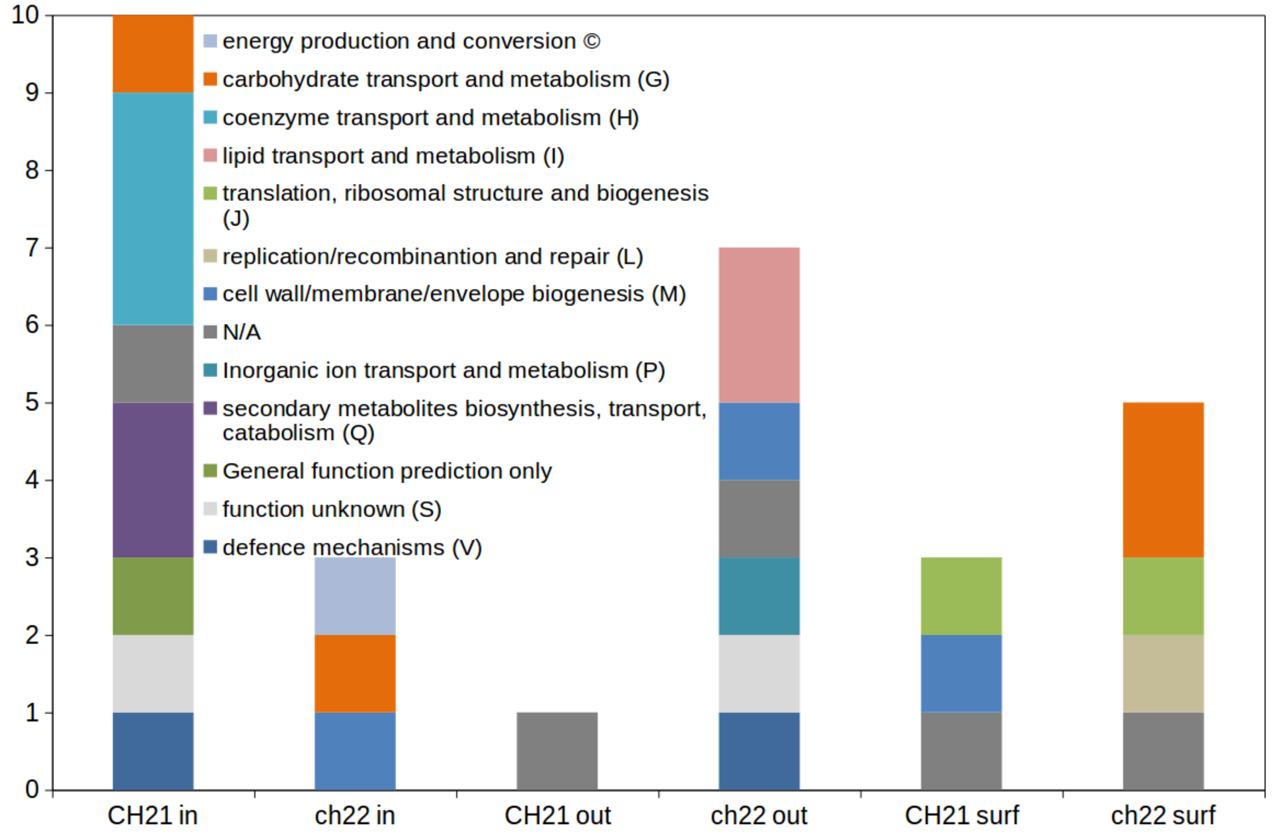

Suppl. fig. 4. Distribution of functional categories of Clusters of Orthologous Groups (COGs) within differentiating proteins identified during analysis of intracellular (in), extracellular (out) and cell membrane/wall-associated (surf) proteome.

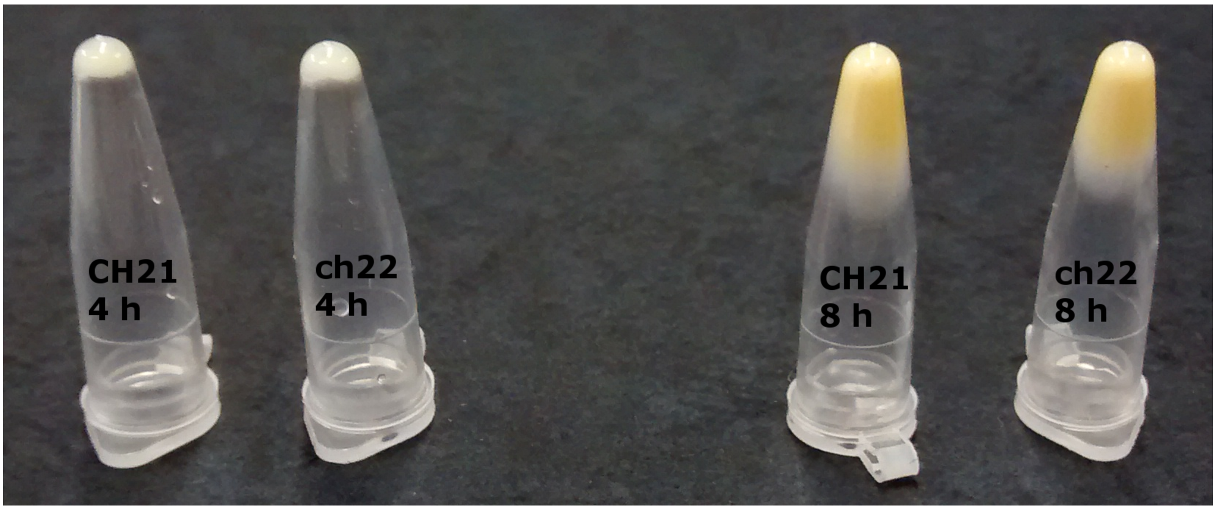

Suppl. fig. 5. Assessment of pigmentation of strains CH21 and ch22. Bacteria were cultured in TSB medium and samples were taken 4 and 8 hours after inoculation, centrifuged, washed with PBS, centrifuged again, and the obtained biomass was photographed.

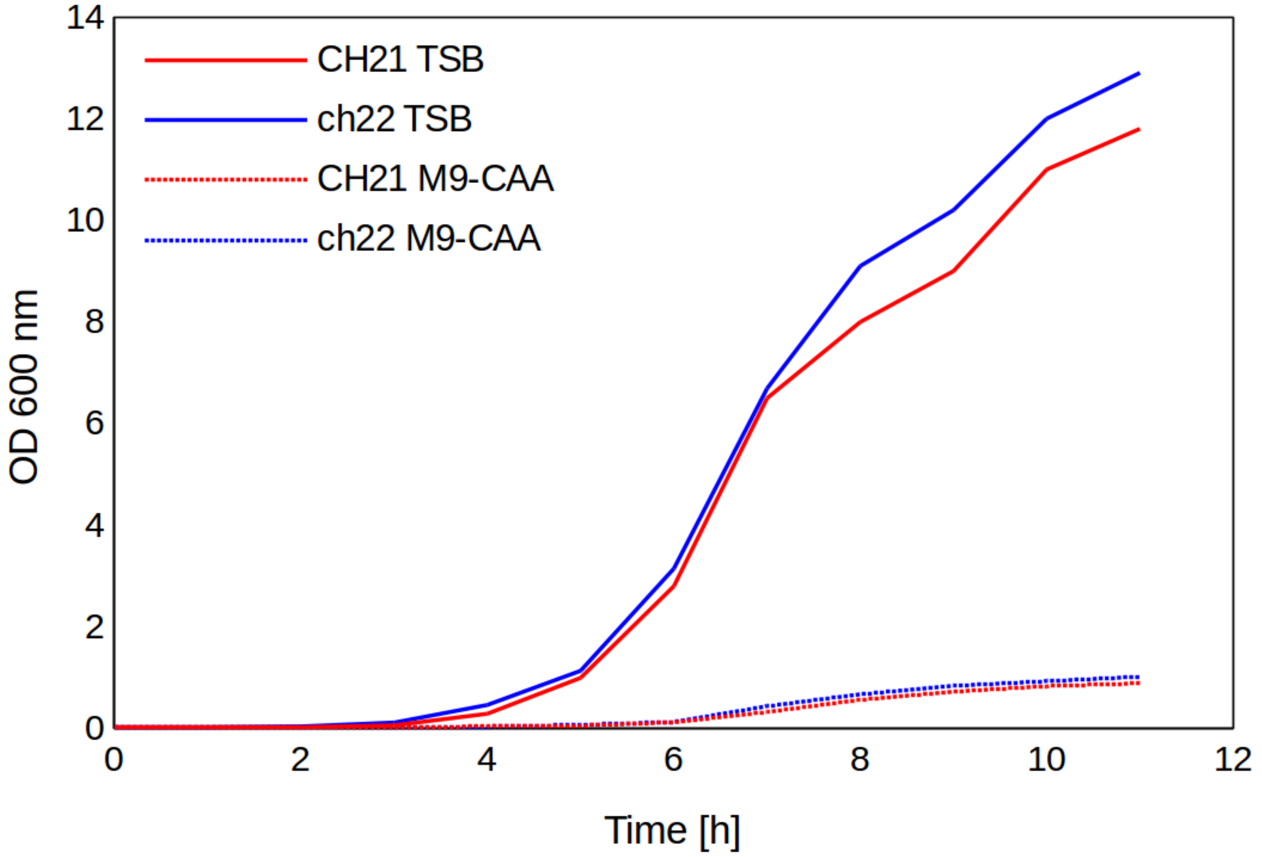

Suppl. fig. 6. Growth curves of CH21 and ch22 grown in rich (TSB) and minimal (M9-CAA) medium.
